# Influenza vaccine is able to prevent neuroinflammation triggered by H7N7 IAV infection

**DOI:** 10.3389/fphar.2023.1142639

**Published:** 2023-03-29

**Authors:** Luisa Demuth, Melanie Ohm, Kristin Michaelsen-Preusse, Kai Schulze, Peggy Riese, Carlos A. Guzmán, Martin Korte, Shirin Hosseini

**Affiliations:** ^1^ Department of Cellular Neurobiology, Zoological Institute, Technische Universität Braunschweig, Braunschweig, Germany; ^2^ Helmholtz Centre for Infection Research, Department of Vaccinology and Applied Microbiology, Braunschweig, Germany; ^3^ Helmholtz Centre for Infection Research, Neuroinflammation and Neurodegeneration Group, Braunschweig, Germany

**Keywords:** inactivated influenza vaccine, influenza infection, neuroinflammation, microglia, dendritic spine

## Abstract

Influenza A virus (IAV) subtypes are a major cause of illness and mortality worldwide and pose a threat to human health. Although IAV infection is considered a self-limiting respiratory syndrome, an expanded spectrum of cerebral manifestations has been reported following IAV infection. Neurotropic IAVs, such as the H7N7 subtype, are capable of invading the central nervous system (CNS) and replicating in brain cells, resulting in microglia-induced neuroinflammation. Microglial cells, the brain’s resident immune cells, are instrumental in the inflammatory response to viral infection. While activation of microglia is important to initially contain the virus, excessive activation of these cells leads to neuronal damage. Previous studies have shown that acute and even long-term IAV-induced neuroinflammation leads to CNS damage. Therefore, the search for possible preventive or therapeutic strategies is of great importance. In this study, we investigated the potential effect of vaccination against acute neuroinflammation induced by H7N7 infection and subsequent neuronal damage in the hippocampus, a particularly vulnerable brain region, comparing young and aged mice. Immunosenescence is one of the striking pathophysiological changes during mammalian aging that leads to “inflammaging” and critically limits the protection by vaccines in the elderly. The results suggest that formalin-inactivated H7N7 vaccine has a preventive effect against the inflammatory responses in the periphery and also in the CNS after H7N7 infection. Cytokine and chemokine levels, increased microglial density, and cell volume after H7N7 infection were all attenuated by vaccination. Further structural analysis of microglial cells also revealed a change in branching complexity after H7N7 infection, most likely reflecting the neuroprotective effect of the vaccination. In addition, synapse loss was prevented in vaccinated mice. Remarkably, engulfment of post-synaptic compartments by microglia can be proposed as the underlying mechanism for spine loss triggered by H7N7 infection, which was partially modulated by vaccination. Although young mice showed better protection against neuroinflammation and the resulting deleterious neuronal effects upon vaccination, a beneficial role of the vaccine was also observed in the brains of older mice. Therefore, vaccination can be proposed as an important strategy to prevent neurological sequelae of H7N7 infection.

## 1 Introduction

In recent decades, several respiratory viruses have emerged that pose a serious threat to global health and economies. Among these viruses, influenza A virus (IAV) is a major respiratory tract pathogen that causes a large number of deaths and hospitalizations worldwide. Infection with IAV results in significant cell death in the upper and lower respiratory tract and lung parenchyma. However, depending on the IAV subtypes and host immune response, the severity of IAV infection can vary from a mild course to massive complications such as pneumonia (Krammer et al., 2018; Flerlage et al., 2021). While seasonal IAV strains (i.e., H3N2 and H1N1) circulate in the population and cause annual epidemics, influenza pandemics occur when cross-species transmission gives rise to new IAV subtypes that can easily infect humans and spread worldwide (Ludlow et al., 2016).

Four global pandemics of influenza infections have been reported in the last century, including the 1918 H1N1 “Spanish flu,” the 1957 H2N2 “Asian flu,” the 1968 H3N2 “Hong Kong flu,” and the 2009 H1N1 “Swine flu.” The 1918 Spanish flu was the most severe pandemic in recent history. Unexpectedly, IAV infections from this deadly pandemic onward have been associated with a plethora of neurological complications, such as encephalitis lethargica, sleep disorder narcolepsy, febrile seizures, meningitis, encephalitis, encephalopathies and post-encephalitic parkinsonism (Reid et al., 2001; Ludlow et al., 2016). In addition to acute neurological complications, IAV infections have also been associated with the progression of neurodegenerative diseases (Cocoros et al., 2021). Furthermore, the coincidence of maternal influenza infection and the prevalence of schizophrenia and bipolar disorder in the offspring has been reported (Brown and Derkits, 2010; Kępińska et al., 2020).

Although the involvement of IAV infections in many central nervous system (CNS) manifestations is likely and has even been noted in several patients (Ekstrand, 2012; Shah et al., 2014), the pathogenesis of IAV-induced CNS disorders remains largely unclear.

Some of the IAV strains defined as neurotropic (i.e., H7N9, H7N7, and H5N1) are capable of infecting neuronal cells and causing severe and sometimes fatal brain inflammation (encephalitis) (Ludlow et al., 2016). The route by which IAVs invade the CNS has not been fully elucidated, but several studies suggest that IAVs may enter the CNS primarily directly from the nasal cavity *via* the olfactory nerve (van Riel et al., 2014). As a result of CNS invasion, neurons and adjacent glial cells become infected and proinflammatory cytokines and chemokines are released far beyond homeostatic levels (Wang et al., 2016; Klein et al., 2019). Subsequently, activation of microglia, the resident immune cells of the brain, can be a double-edged sword, initially protecting the brain from viral invasion, but also leading to neuronal damage through direct cytolytic actions, neuronal priming, or concomitant inflammatory responses (Jang et al., 2012; Sadasivan et al., 2015).

Microglia are the major producers of the proinflammatory cytokines tumor necrosis factor alpha (TNFα), interleukins (IL)-6, IL-1 and complement factor 3 (C3) in the brain. These factors are described as important triggers of acute seizures in viral encephalitis, as excessive production of these factors can lead to neuronal hyperexcitability (Cusick et al., 2013). Microglia can also produce type I and II interferons (IFN) as well as C-C motif ligand 2 (CCL2) and CCL5, which can have direct neurotoxic effects (Quick et al., 2014). Apart from the proinflammatory cytokines released by microglia causing neuronal damage, microglia might also be directly involved in the neurological manifestations induced by viral infection through direct excessive synaptic pruning, their physiological roles during brain development (Paolicelli et al., 2011; Chen et al., 2019). For instance, experimental studies using West Nile virus infected mice have shown that complement components mediate loss of presynaptic compartments in the hippocampus, leading to impaired spatial learning (Garber et al., 2019). During viral infection, C3 localizes to presynaptic terminals, prompting microglia expressing C3a receptor to congregate around neurons, exert phagocytosis, and engulf the presynaptic compartment. This might be a protective mechanism following neurotropic viral infection as this neuron-microglia interaction leads to the removal of pre-synapses which can prevent trans-synaptic spread of the virus and terminate abnormal signaling from the infected neurons (Vasek et al., 2016). In contrast, Zika virus infection resulted in loss of postsynaptic terminals, which may be a prelude to neuronal loss (Garber et al., 2019).

Experiments in animal models have previously shown that IAV infections trigger neuroinflammation in the acute phase of the disease, including activation of microglia and release of proinflammatory cytokines (Jang et al., 2012; Jurgens et al., 2012; Sadasivan et al., 2015; Hosseini et al., 2018). Even sublethal infection with a neurotropic H7N7, as well as non-neurotropic H1N1 and H3N2 IAV strains, led to an extensive peripheral immune response, followed by a mirror inflammatory response in the CNS and changes in microglial activation status and astrocyte reactivity as crucial regulators of innate and adaptive immune responses in the CNS (Hosseini et al., 2018). Therefore, IAV-associated CNS damage could be an indirect effect of infection attributable to systemic cytokines, a direct effect of virus entry into the CNS, or a combination of both (Kuiken and Taubenberger, 2008).

Evidence accumulates that susceptibility to pulmonary infections caused by respiratory viruses increases with age (Wu et al., 2021). Indeed, a progressive decline in the integrity of the immune system, termed immunosenescence, is one of the prominent physiological changes during mammalian aging that causes “inflammaging.” This describes the close relationship between low-grade chronic inflammation and aging, which has been associated with a wide range of age-related disorders in various organs, including the brain (Gabuzda and Yankner, 2013).

Since the brain, particularly the hippocampus, is highly susceptible to inflammatory responses and has limited regenerative capacity (Chesnokova et al., 2016), disruptions to its homeostasis can have profound consequences for important cognitive functions, severely affecting the quality of life in patients. Therefore, it is of great importance to find an effective way to prevent harmful neurological consequences of IAV infections of the brain in the acute phase of the disease to avoid long-term consequences (Jang et al., 2012; Hosseini et al., 2018).

Yet, the development of effective and promising therapeutics is hampered by several factors. Due to the unique properties of the CNS microvasculature, many large and polar molecules cannot enter the CNS, limiting the selection of promising therapeutic molecules (Daneman and Prat, 2015). In addition, the timing of drug administration most likely need to be very specific, whereas the exact time point of infection in humans remains unclear in most cases. For these reasons, prevention of neurological sequelae by vaccination is the most promising and valuable approach.

Therefore, this study investigated the effect of a formalin-inactivated H7N7 vaccine against acute neuroinflammation and subsequent neuronal damage in both young and elderly individuals. The results of this study provide insight into the beneficial effects of vaccination on attenuating excessive neuroinflammatory responses and neuronal damage triggered by infection and could be proposed as a preventive strategy against neurological manifestations caused by IAV infection, although this beneficial effect was more pronounced in young adults.

## 2 Material and methods

### 2.1 Animals

For this study, 2-month-old (young) and 15-month-old (elderly) female C57BL/6JRj mice were purchased from Janvier Labs, France. Animals were maintained under specific pathogen-free (SPF) conditions on a 12-h light-dark cycle with *ad libitum* access to water and food at the central mouse facility of the Helmholtz Centre for Infection Research, Braunschweig, Germany. All experimental procedures were reviewed and approved by the local committees of the Helmholtz Centre for Infection Research and TU Braunschweig, Germany, and the authorities (LAVES, Oldenburg, Germany; approval number: 18/2968) according to the national guidelines of the animal welfare law in Germany.

### 2.2 Influenza vaccination

Stocks of rSC35M, mouse-adapted A/Seal/Mass/1/80 (H7N7) influenza A virus were obtained from Gülsah Gabriel, Heinrich-Pette Institute, Hamburg, Germany (Gabriel et al., 2005) and propagated in MDCK (Madin–Darby Canine Kidney) cell cultures as previously described (Wilk and Schughart, 2012). To prepare the formalin-inactivated virus vaccine, viruses were resuspended in phosphate-buffered saline (PBS). Then, the purified viruses were treated with 0.1%–0.2% formalin at 4°C for 1 week. Inactivation of the virus in each vaccine preparation was confirmed by the absence of detectable hemagglutination activity after inoculation of the treated materials into embryonated eggs.

**FIGURE 1:**
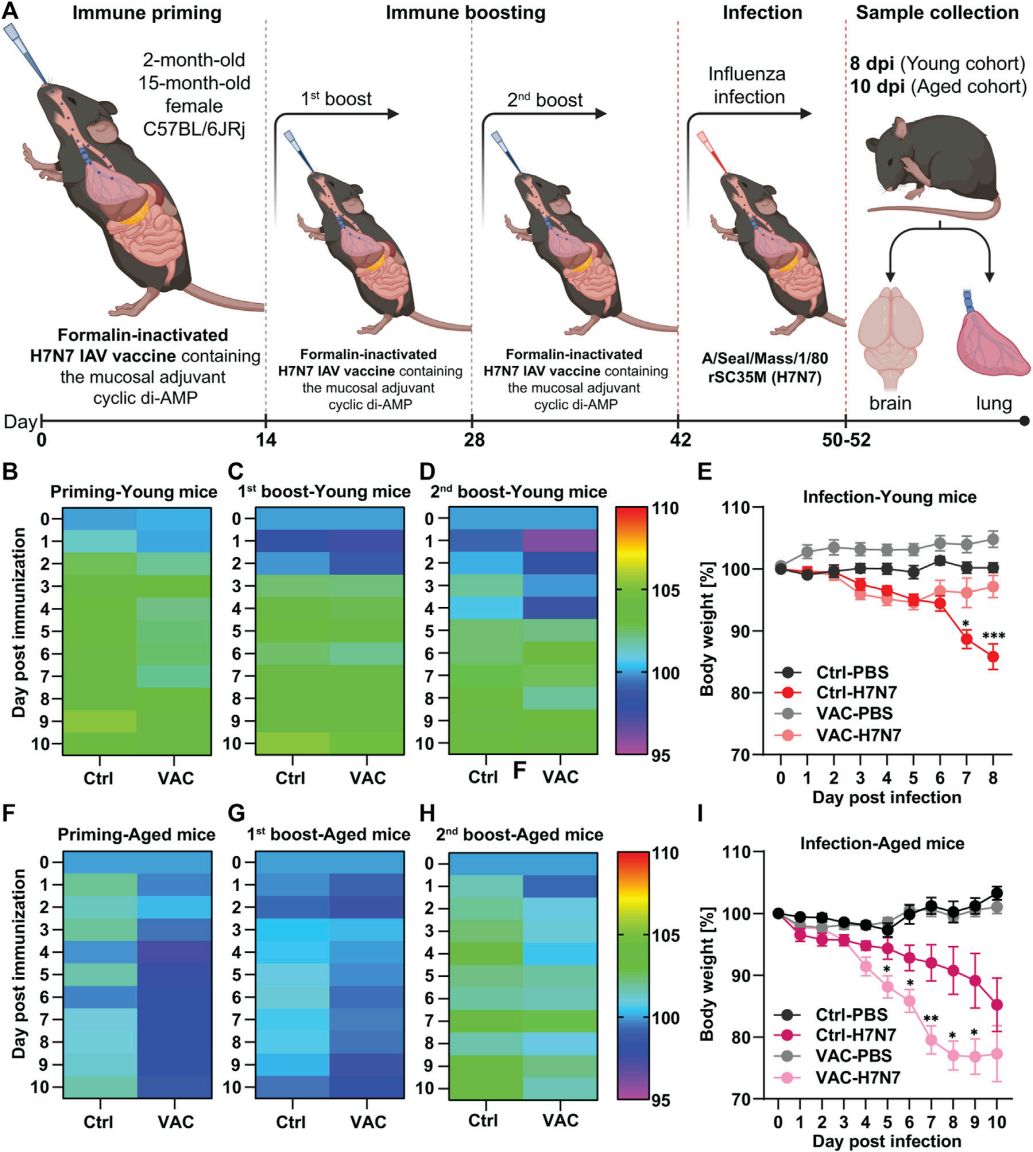
Immunization of young and old C57BL/6JRj mice with formalin-inactivated H7N7 IAV vaccine against H7N7 IAV infection. **(A)** Schematic overview of the immunization schedule for young and old mice followed by infection with 10 FFU influenza A/Seal/Mass/1/80 rSC35M (H7N7) virus. During the peak of infection, brain and lungs were harvested from both young (8dpi) and old (10 dpi) mice for further analysis (Created with BioRender.com). In 2-month-old mice **(B)** after immune priming, **(C)** after first immune boost, and **(D)** after second immune boost, the body weight of the animals was comparable and, after an initial decrease, steadily increased slightly in the following days, although the initial decrease was more pronounced in the vaccinated animals (*n* = 28–32 animals per group). **(E)** While the body weight loss was relatively constant after PBS inoculation in both the Ctrl and VAC groups, the body weight of the mice infected with the H7N7 IAV began to decrease uniformly by day 6 post-infection. Between days 6 and 8 post-infection, the body weight of the vaccinated animals returned to the control level, while the Ctrl mice infected with H7N7 IAV lost even more weight (*n* = 10–14 animals per group). In 15-month-old mice, **(F)** body weight loss after immune priming was more pronounced in the vaccinated group. This was also the case **(G)** after the first immune boost and **(H)** after the second immune boost, but not as pronounced as with immune priming (*n* = 13–15 animals per group). **(I)** After H7N7 IAV infection, both Ctrl-PBS and VAC-PBS showed similar changes in body weight. However, both control and vaccinated groups infected with H7N7 IAV showed body weight loss from day 4, which was even significantly greater in the previously vaccinated mice (*n* = 6–8 animals per group). Data are presented as mean ± SEM and were analyzed with the repeated measures two-way ANOVA followed by Fisher’s LSD test; *p < 0.05, **p < 0.01, and ***p < 0.001.

For immunization, mice were randomly divided into two groups and then individually exposed to isoflurane in 100% oxygen using an isoflurane anesthesia machine for light anesthesia. Then, the control animals were inoculated intranasally with 20 μL Ampuwa^®^ (water for injection) and the immunized animals were administered intranasally a dose of 8.4 µg per mouse of formalin-inactivated H7N7 IAV vaccine with 10 μg/μL of mucosal adjuvant cyclic di-AMP in 20 µL Ampuwa^®^ as immune priming. The first immune boost with the same procedures was administered on day 14 after priming and the second immune boost was administered on day 28 (Figure 1A). Body weight and physical appearance, including spontaneous behavior, posture, fur and behavior on provocation, were assessed for ten consecutive days after each vaccination.

### 2.3 Influenza infection

For the infection procedure, 14 days after the last vaccination (Figure 1A) both young and elderly mice were first anesthetized with an intraperitoneal (i.p.) injection of ketamine-xylazine solution (85% NaCl (0.9%), 10% ketamine (100 mg/mL), 5% xylazine (20 mg/mL); 10 mL per 1 kg body weight) and then intranasally infected with a sublethal low dose of 10 FFU (focus-forming units) of rSC35M, mouse-adapted A/Seal/Mass/1/80 (H7N7) influenza A virus in 20 μL sterile PBS 1X (Hosseini et al., 2018). Body weight and physical appearance, including spontaneous behavior, posture, fur, and behavior on provocation, were assessed until the acute phase of infection, which was 8 days in the young cohort and 10 days in the aged cohort. Percent body weight loss was calculated compared with body weight at day 0 (prior to infection). Mice with body weight loss greater than 30% or severe physical complications were euthanized for ethical reasons. In the uninfected control groups, mice were intranasally inoculated with 20 μL sterile PBS 1X (Hosseini et al., 2018).

### 2.4 Enzyme-linked immune sorbent assay (ELISA)

ELISA assays were performed to quantify the levels of chemokines (CCL2) and cytokines (IFNγ, IL1β, IL-6, IL-10, and TNFα) in the supernatants of the lungs and cerebral hemispheres of all experimental groups. For this purpose, both young and aged cohorts of female C57BL/6JRj mice were deeply anesthetized with CO_2_ and killed by decapitation 8 or 10 days post-infection (dpi). The lungs and right hemisphere of the brain were harvested. For protein isolation, tissues were homogenized with an Eppendorf-fitting pestle in 1 mL (lung) or 400 μL (cerebral hemisphere) of STKM lysis buffer containing (in mM): 250 sucrose, 50 Tris-HCl, 25 KCl, and 5 MgCl_2_ and protease inhibitor mixture (Roche cOmplete™ Protease Inhibitor Cocktail tablet). Samples were centrifuged at 4°C for 10 min at 13,000 × g. The supernatant was collected and stored at −80°C until assayed. Mouse CCL2/JE/MCP-1 DuoSet (DY479), Mouse IFN-gamma DuoSet (DY485), Mouse IL-1beta/IL-1F2 DuoSet (DY401), Mouse IL-6 DuoSet (DY406), Mouse IL-10 DuoSet (DY417), and Mouse TNF-alpha DuoSet (DY410) ELISA kits (R&D SYSTEMS) were used to determine chemokine and cytokine levels according to the manufacturer’s recommendations. Absorbance at 450 nm was measured using a Tecan Sunrise microplate reader connected to Magellan software. Finally, the measured optical density of the reaction was compared with the optical density of the known standard samples to determine the protein concentration in the samples.

### 2.5 Immunohistochemistry

To study hippocampal microglial cells, left cerebral hemispheres from young and aged cohorts were isolated 8 or 10 days after H7N7 IAV infection and fixed in 4% paraformaldehyde (PFA) for 24 h. The cerebral hemispheres were then cryoprotected in 30% sucrose solution in PBS 1X for 24 h and stored in Tissue-Tek optimal cutting temperature compound (Hartenstein Laborversand) at −70°C as previously described (Hosseini et al., 2018; Hosseini et al., 2021b). Frozen brain hemispheres were cut into 25 µm thick slices using a Leica 2800E Frigocut cryostat microtome. Then, six consecutive sections per mouse were transferred to the 24-well plate for free-floating immunohistochemical experiments. The sections were washed twice with PBS 1X for 2 min each and three times with 0.1% Triton X-100 for 5 min each, while the plate was always kept on the shaker. This was followed by a 1-h incubation in the blocking solution containing 0.3% Triton X-100, 5% goat serum, 5% donkey serum, and 5% bovine serum albumin (BSA) at room temperature (RT) on the shaker. The sections were then incubated overnight at 4°C with the primary antibodies diluted in blocking solution, including rabbit polyclonal anti-IBA1 (1:1,000, Synaptic Systems—RRID: AB_10641962), rat anti-mouse CD107a (LAMP-1; 1:500, BD Pharmingen™—RRID: AB_2134499), chicken polyclonal anti-Homer-1 (1:500, Synaptic Systems—RRID: AB_2631222) and mouse monoclonal anti-GFAP (1:1,000, Sigma-Aldrich - RRID: AB_477010), on the shaker.

The next day, the plate was incubated on the shaker for 30 min at RT and then washed three times with PBS 1X for 10 min each time. In the next step, the sections were incubated with the secondary antibodies including Cy™3 AffiniPure goat anti-rabbit IgG (H + L) (1:500, Jackson Immuno Research - RRID: AB_2338006), Cy™5 AffiniPure goat anti-rat IgG (H + L) (1:500, Jackson Immuno Research - RRID: AB_2338264), Alexa Fluor^®^ 488 AffiniPure donkey anti-chicken IgY (IgG) (H + L) (1:500, Jackson Immuno Research - RRID: AB_2340375) and Cy™3 AffiniPure goat anti-mouse IgG (H + L) (1:500, Jackson Immuno Research—RRID: AB_2338686) in 0.05% Triton X-100 and PBS 1X for 2 h at RT on the shaker in the dark. They were then washed three times with PBS 1X for 10 min each, incubated with 4′,6-diamidino-2-phenylindole (DAPI) (1:1,000, Sigma-Aldrich) for 5 min, and then washed again six times with PBS 1X for 5 min each. Finally, the sections were mounted on glass slides in fluorogel embedding medium (Electron Microscopy Sciences, Hatfield, PA).

### 2.6 Golgi-Cox staining

To examine hippocampal neuron morphology, Golgi-Cox staining was performed using the FD Rapid GolgiStain™ Kit (FD Neuro-technologies, Inc.) according to the manufacturer’s protocol. Eight or 10 days after H7N7 IAV infection, after killing with CO_2_ and decapitation, the right cerebral hemispheres of the different experimental groups were incubated in the Golgi solution mixture, while the left ones were used for immunohistochemistry. Before sectioning, the cerebral hemispheres were embedded in 2% agar. Coronal sections of the hemispheres with a thickness of 150 µm were cut using a Leica Vibratome (VT 1000S) and mounted on gelatin-coated slides. In the following steps, the sections were further processed for signal development according to the kit manufacturer’s protocol. Finally, the sections were mounted using Permount (Thermo Fisher Scientific).

### 2.7 Imaging and image analysis

#### 2.7.1 Imaging and quantification of microglial cell density

Immunohistochemically stained sections were first imaged using an Apotome microscope (Imager.M2 AXIO, ZEISS) with the ×20 objective (N.A. 0.8). Only DAPI and Cy3 channels (for detection of IBA-1 or GFAP positive cells) were used, and Z-stacks with intervals of 1 µm were imaged from five to six hippocampal slices per animal. For each section, the CA1 and *dentate gyrus* subregions of the hippocampus were imaged to determine microglial density. Analysis of these images was performed blindly using Fiji software (BioVoxxel). For this purpose, eight slices were selected from the Z-stacks in the center of the stack and flattened in 2D using the “Z-Project” tool with the “maximum intensity” setting. The two channels (Cy3 and DAPI) were then merged back together using the “Color” and “Merge Channels” tools. All microglial cells in the image were manually counted using the “Multiple Points” tool. Cell density in the image area (number of cells/mm^2^) was then calculated in Excel (Microsoft). Fiji was also used to examine the fluorescent intensity of GFAP staining as a marker of astrocytic activation in the CA1 and DG subregions of the hippocampus. All data were normalized within each staining to the mean of the control (unvaccinated) group inoculated with PBS.

#### 2.7.2 Single cell imaging and analysis of microglia

Individual microglial cells were imaged from the triple-stained sections (IBA-1/LAMP-1/Homer-1). A confocal laser scanning microscope (cLSM, Olympus) was used to image Z-stacks of microglial cells in 0.35-µm increments with a ×40 UPLFLN oil objective (N.A. 1.30) and ×6 zoom. The final pixel size was 0.103 µm × 0.103 µm. For each animal, Z-stacks of three randomly selected individual microglial cells in the CA1 and *dentate gyrus* subregions of three sections were acquired. Before analysis in IMARIS (Bitplane), images were deconvoluted by blind 3D deconvolution in AutoQuantX (Adobe Systems GmbH).

In IMARIS, the surface of microglial cells was modeled using IBA-1 staining (surface detail: 0.2 µm). Then, within the constructed microglial surface, the LAMP-1 positive signals were masked to model the surface of the LAMP-1 vesicles (surface detail: 0.2 µm). The Homer-1 spots in the LAMP-1 vesicles within the microglial cells were labeled with the spot function (spot diameter: 0.5 µm). In addition, the microglia structure was accessed by first masking the IBA-1 signal into the constructed IBA-1 cell surface. The “Add New Cell” tool was used to model the cell soma (filter width: 1 μm, sphere diameter: 0.8 µm) and “Filament Analysis” was used to access the microglial branching complexity (largest diameter: 5 μm, thinnest diameter: 0.3 µm, sphere region diameter: 15 µm). Various parameters such as IBA-1 volume in µm^3^, LAMP-1 in IBA-1 volume in µm^3^, number of Homer-1 puncta in LAMP-1, number of branching points, and size of microglial somas in µm^3^ were recorded and transferred to Excel (Microsoft). All data were normalized to the mean of the PBS-inoculated control group (unvaccinated) within each staining.

#### 2.7.3 Image analysis of the Golgi-Cox stain

After a drying period of at least 2 weeks, Golgi-stained sections were imaged using a ZEISS microscope equipped with an Apotome module and a ×63 objective (N.A. 1.4, oil). Z-stacks in 0.3 µm steps were imaged from the secondary apical dendrites of the CA1 pyramidal neuron and from the dendrites of the granule cells in the superior leaflet of the *dentate gyrus*. At least 8 dendrites longer than 60–70 μm, at least 40–50 µm from the cell soma, were imaged per region and animal. Spine density per µm of imaged dendrites was analyzed manually using Fiji software (BioVoxxel). All slides were coded and analysis was performed blindly.

### 2.8 Statistical analysis

Data were presented as mean ± SEM and analyzed and plotted with GraphPad Prism 9 (GraphPad Software, Inc., United States). Differences between experimental groups were subjected to an ordinary two-way ANOVA (two variable factors: vaccination and infection). Fisher’s LSD multiple comparison was used as a post-hoc test. The minimum significance value was considered as *p* < 0.05. The minimum number of animals in all experiments was calculated *a priori* using G∗Power 3.1.9.4 software (Heinrich Heine College Duesseldorf, Germany). The “n” of the different experimental groups is indicated in the respective figure legends. All experiments were evaluated in a strictly blind fashion.

## 3 Results

### 3.1 Intranasal immunization with formalin-inactivated H7N7 vaccine against H7N7 infection in mice

Both young (2-month-old) and old (15-month-old) mice were randomly divided into two groups. One group was immunized intranasally three times at 14-day intervals with a formalin-inactivated H7N7 vaccine containing a cyclic di-AMP adjuvant (VAC), while the other group served as a control (Ctrl) and was inoculated with Ampuwa^®^ (water for injection) instead. Fourteen days after the second boost, more than half of each group was intranasally infected with the H7N7, while the others were inoculated with PBS, resulting in four different experimental groups: Ctrl-PBS, Ctrl-H7N7, VAC-PBS, and VAC-H7N7. Over a period of 10 days after each vaccination, the body weight of the animals was monitored as well as after H7N7 infection until the mice were killed in the acute phase of infection (8 dpi for the young cohort and 10 dpi for the older cohort) (Figure 1A). The period of 8 days for the young cohort and 10 days for the older cohort was chosen based on previous observations that maximum body weight loss occurs during the respective days after H7N7 infection (Hosseini et al., 2018).

In 2-month-old mice after the first vaccination (immune priming), the body weight of the vaccinated animals was not significantly different from that of the control animals and increased by approximately 5% over the following 10 days (Figure 1B). In the first 2 days after the first immune boost, both vaccinated and control mice showed a slight decrease in body weight, which was followed by an increase of about 5% in both groups (Figure 1C). This observation intensified somewhat after the second immune boost in the vaccinated group, in which a stronger decrease in body weight was observed for several days, followed by an increase in body weight starting on day 5 (Figure 1D). Thus, it appears that immune responses were stronger after the second boost, resulting in greater body weight loss during the first few days.

After infection, the body weight of control mice inoculated with PBS was relatively stable, whereas in the group that had been previously vaccinated and then inoculated with PBS, body weight increased slightly in the 8 days after inoculation. However, the difference between the groups was not statistically significant (Figure 1E). Compared with the control mice, the body weight of the two groups infected with H7N7 was stable until the second day after infection. Then, H7N7 infection caused a decrease in body weight in both control and vaccinated mice until day 6 post-infection. From day 6, the body weight of the vaccinated animals increased again and returned to the control level on day 8 post-infection. In contrast, the H7N7 infected control animals showed an increasing trend of weight loss that was significant compared to the vaccinated H7N7 infected mice on day 7 (*p* = 0.01) and day 8 (*p* = 0.0003) post H7N7 infection (Figure 1E). While in these young mice, the survival rate of control animals after H7N7 infection to peak infection (8 dpi) was approximately 92%, 100% of vaccinated mice survived to day eight post-infection.

However, in the older cohort, the results showed that body weight decreased slightly after the first vaccination (immune priming), whereas this was not the case in the control group (Figure 1F). Intrestingly, there were no significant differences between the control and vaccinated groups after the first and second immune boosts, although body weight loss was always more pronounced in the vaccinated group (Figures 1G, H). Maybe the response to the vaccine was not very effective in the older cohort due to immunosenescence. This was even more evident after H7N7 infection (Figure 1I). Both groups inoculated with PBS (Ctrl-PBS and VAC-PBS) showed similar changes in body weight. However, from day 4 post-infection, both control and vaccinated groups infected with H7N7 showed a decrease in body weight until day 10 post-infection, when the mice were killed. Surprisingly, the loss of body weight at several days (day 5: *p* = 0.03, day 6: *p* = 0.03, day 7: *p* = 0.009, day 8: *p* = 0.02, day 9: *p* = 0.05) was significantly greater in the vaccinated mice compared with the infected control mice. However, comparison of survival rates between control mice and vaccinated old mice showed that although infection was more severe in the vaccinated mice, a greater proportion of the old mice survived the infection. About 88% of the vaccinated mice survived the H7N7 infection, while the infection in the control mice resulted in a survival rate of 72%, confirming the generally beneficial effect of vaccination. Overall, these results showed that influenza vaccination attenuated the consequences of infection in young mice. This was not the case in older mice, although they had a higher survival rate compared with unvaccinated mice, consistent with observations in young individuals.

### 3.2 Effect of immunization against lung and brain inflammation induced by H7N7 infection

**FIGURE 2:**
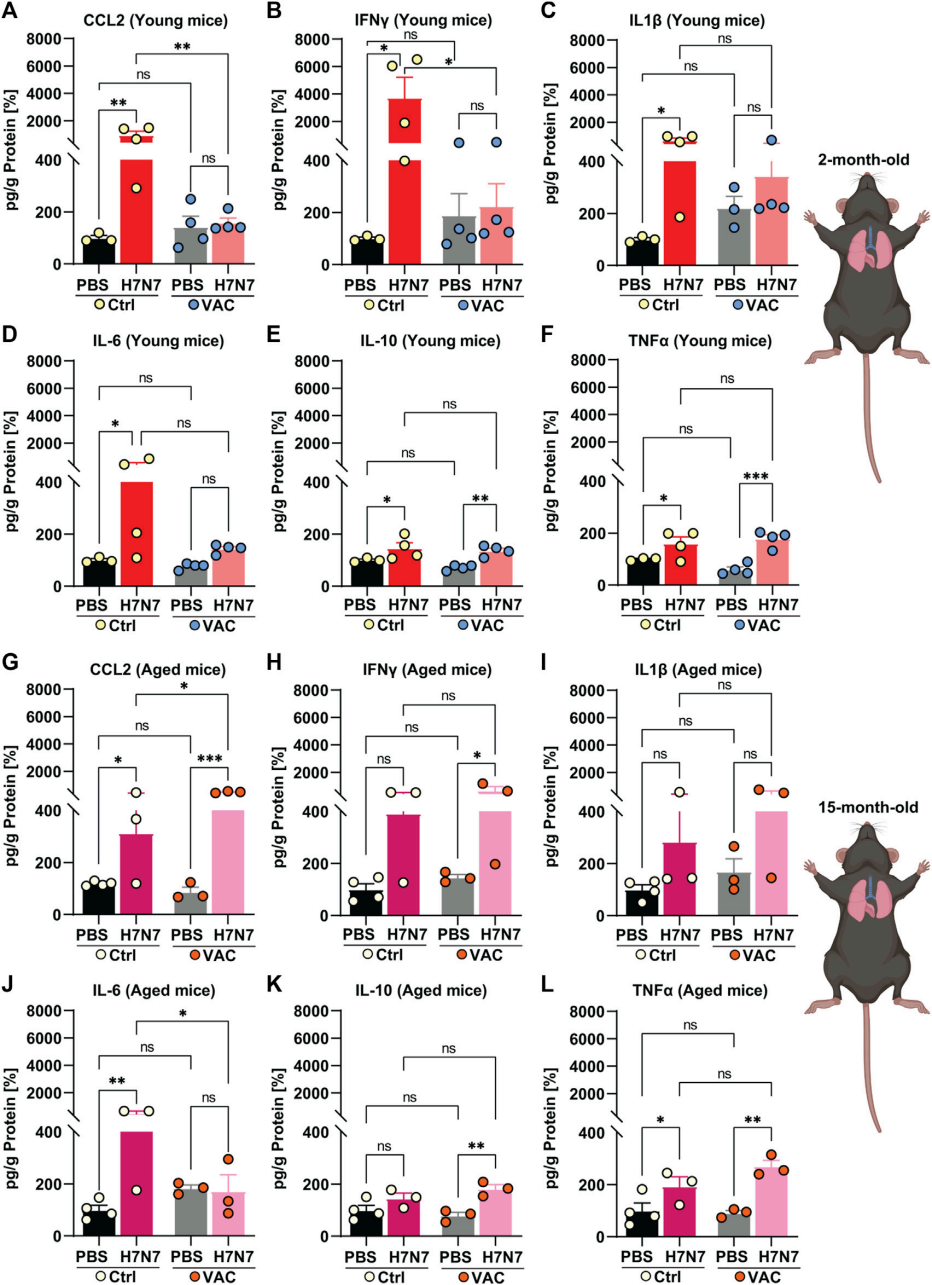
Effects of vaccination on the release of cytokines and chemokines in the lung induced by H7N7 IAV infection. In the lungs of young mice, levels of **(A)** CCL2 and **(B)** IFNγ increased after H7N7 IAV infection, whereas vaccination prevented this increase and resulted in a significant decrease in the release of these mediators in infected mice. H7N7 IAV infection led to a significant increase in the levels of **(C)** IL1β and **(D)** IL-6 only in the unvaccinated mice. H7N7 IAV infection resulted in increased levels of **(E)** IL-10 and **(F)** TNFα in the lungs of young mice, regardless of whether they were vaccinated or not (*n* = 3–4 animals per group). In the lungs of older mice, H7N7 IAV infection resulted in increased release of **(G)** CCL2, **(H)** IFNγ, and **(I)** IL1β independent of vaccination. CCL2 levels were even higher in the lungs of infected mice that had been previously vaccinated. **(J)** IL-6 levels increased only in the lungs of old unvaccinated mice after H7N7 IAV infection. **(K)** IL-10 levels increased only in the lungs of vaccinated mice after infection. H7N7 IAV infection resulted in increased **(L)** TNFα levels in the lungs of aged mice, whether vaccinated or not (*n* = 3–4 animals per group). Data are presented as mean ± SEM and were analyzed with an ordinary two-way ANOVA followed by Fisher’s LSD test; *p < 0.05, **p < 0.01, and ***p < 0.001.

**FIGURE 3:**
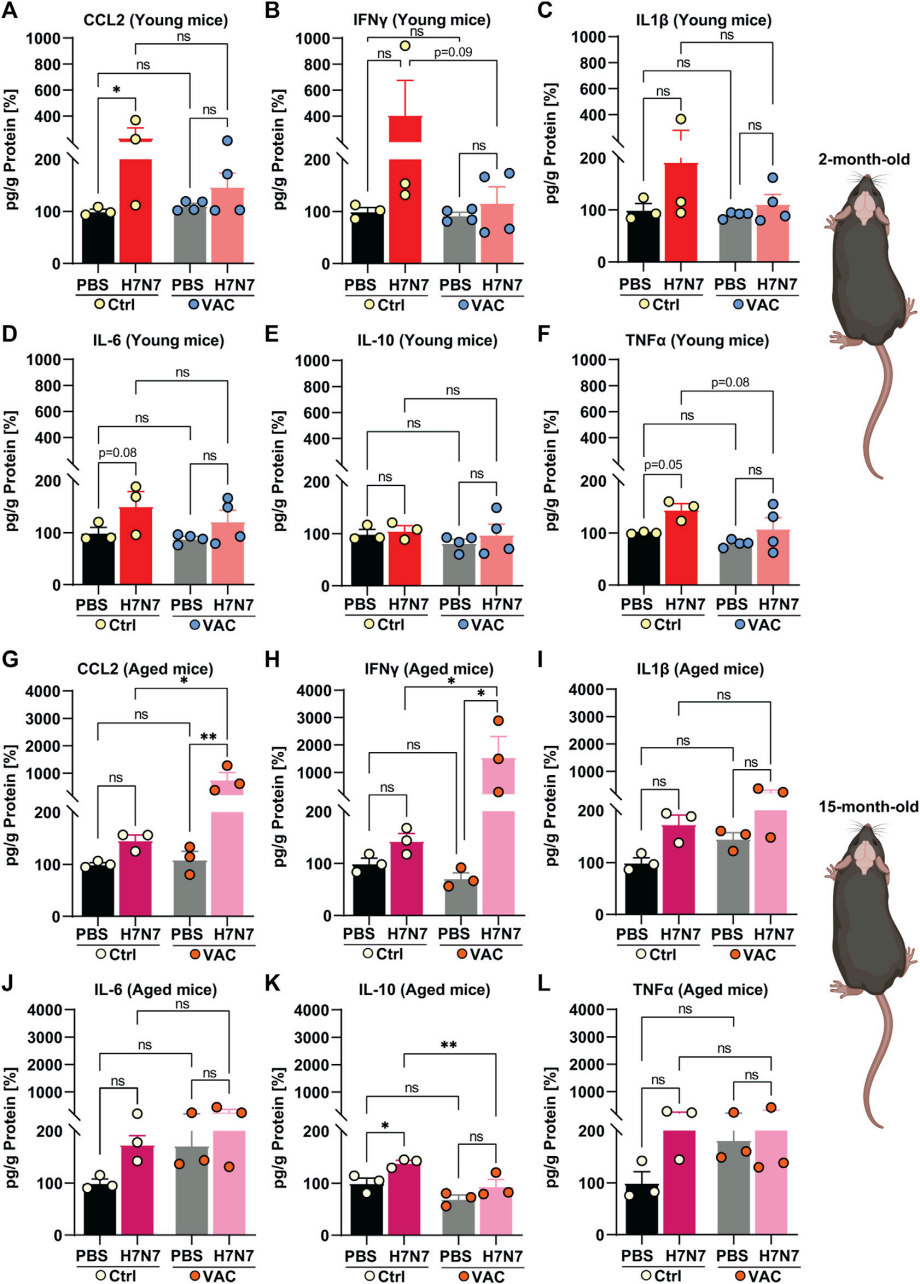
Effects of vaccination on the release of cytokines and chemokines in the brain induced by H7N7 IAV infection. In the brains of young mice, levels of **(A)** CCL2, **(B)** IFNγ, **(C)** IL1β, **(D)** IL-6, and **(F)** TNFα increased after H7N7 IAV infection, whereas vaccination prevented this increase and resulted in a lower release of these mediators in the brain of infected mice. **(E)** IL-10 was not increased in the brains of H7N7 IAV infected mice, regardless of whether they were vaccinated or not (*n* = 3–4 animals per group). In the brains of older mice, H7N7 IAV infection resulted in increased release of **(G)** CCL2, **(H)** IFNγ, and **(I)** IL1β independent of vaccination. CCL2 and IFNγ levels were even higher in the brains of infected mice that had been previously vaccinated. Levels of **(J)** IL-6 and **(L)** TNFα were higher in the brains of unvaccinated mice after H7N7 IAV infection than in the Ctrl-PBS group. **(K)** IL-10 levels increased only in the brains of unvaccinated mice after H7N7 IAV infection (*n* = 3 animals per group). Data are presented as mean ± SEM and were analyzed with an ordinary two-way ANOVA followed by Fisher’s LSD test; *p < 0.05, and **p < 0.01.

Following infection with various IAV strains, excessive levels of inflammatory mediators and widespread tissue damage can be observed in the lung, a target tissue for respiratory viruses (Jang et al., 2012; Burggraaf et al., 2014; Herold et al., 2015; Taye et al., 2017). Moreover, several studies have shown that neurotropic and even non-neurotropic IAV infections lead to the release of inflammatory mediators in the brain (Jang et al., 2012; Jurgens et al., 2012; Hosseini et al., 2018; Düsedau et al., 2021). Therefore, to determine the inflammatory responses triggered by H7N7 infection and the potential beneficial effects of vaccination, this study quantified the levels of several key cytokines and chemokine in the lung (Figure 2) and brain (Figure 3) of all experimental groups at the peak of infection (8 dpi for the young cohort and 10 dpi for the older cohort) by ELISA.

In 2-month-old mice, infection with H7N7 IAV at day 8 post-infection resulted in an increase in CCL2 [Figure 2A, two-way ANOVA F_infection_(1, 11) = 7.50, *p* = 0.02], IFNγ [Figure 2B, two-way ANOVA F_infection_(1, 11) = 4.86, *p* = 0.04], IL1β [Figure 2C, two-way ANOVA F_infection_(1, 10) = 6.84, *p* = 0.02], and IL-6 [Figure 2D, two-way ANOVA F_infection_(1, 11) = 4.16, *p* = 0.06] in the lungs of unvaccinated mice compared to control mice inoculated with PBS (CCL2: *p* = 0.003, IFNγ: *p* = 0.01, IL1β: *p* = 0.01, IL-6: *p* = 0.04). However, comparisons between vaccinated mice showed that H7N7 infection did not significantly increase levels of CCL2 (Figure 2A, *p* = 0.94), IFNγ (Figure 2B, *p* = 0.97), IL1β (Figure 2C, *p* = 0.52), and IL-6 (Figure 2D, *p* = 0.59) in the lungs compared to vaccinated mice receiving PBS.

Intrestingly, H7N7 infection resulted in significantly higher levels of IL-10 [Figure 2E, two-way ANOVA F_infection_(1, 11) = 17.43, *p* = 0.001] and TNFα [Figure 2F, two-way ANOVA F_infection_(1, 11) = 26.24, *p* = 0.0003] in the lungs of mice, regardless of whether they had been previously vaccinated or not, compared to their respective controls. In young mice, comparison between the two groups inoculated with PBS (Ctrl-PBS and VAC-PBS) showed that immunization *per se* did not significantly alter the levels of these inflammatory mediators, at least at this time point, which was 22 days after the second immune boost (Figures 2A–F). However, after infection with the H7N7, vaccination resulted in significantly lower CCL2 (*p* = 0.003) and IFNγ (*p* = 0.01) levels in the lungs of young mice compared with unvaccinated infected mice (Figures 2A, B).

Surprisingly, aged mice showed different results than young mice. Ten days after H7N7 infection, levels of almost all cytokines and chemokine increased compared with the corresponding PBS-inoculated groups (Ctrl-PBS and VAC-PBS), regardless of prior vaccination (Figures 2G–L).

Indeed, CCL2 [Figure 2G, two-way ANOVA F_infection_(1, 9) = 37.05, *p* = 0.0002], IFNγ [Figure 2H, two-way ANOVA F_infection_(1, 9) = 8.03, *p* = 0.01], and IL1β [Figure 2I, two-way ANOVA F_infection_(1, 9) = 5.21, *p* = 0.04] levels were increased after H7N7 infection, and even this increase was statistically significant in the case of CCL2 (*p* = 0.03) in the vaccinated group compared with unvaccinated mice.

In aged unvaccinated mice, although H7N7 infection increased IL-6 levels (Figure 2J) in the lungs compared to the Ctrl-PBS group (*p* = 0.005), vaccination successfully prevented this H7N7 IAV infection-induced increase (*p* = 0.02).

Moreover, the levels of IL-10 (Figure 2K) were significantly increased only in the lungs of H7N7 IAV infected mice that had been previously vaccinated compared to the VAC-PBS group (*p* = 0.003).

Consistent with the observation in the young mice, H7N7 infection resulted in significantly higher TNFα levels [Figure 2L, two-way ANOVA F_infection_(1, 9) = 24.21, *p* = 0.0008] in the lungs of the mice, whether previously vaccinated or not, compared to their respective controls.

Next, the levels of inflammatory mediators were examined in the brains of young and aged mice in all experimental groups (Figure 3). In the young cohort, when unvaccinated mice were compared, the results showed that infection with H7N7 resulted in higher levels of CCL2 (Figure 3A, *p* = 0.02), IFNγ (Figure 3B, *p* = 0.10), IL1β (Figure 3C, *p* = 0.14), IL-6 (Figure 3D, *p* = 0.08), and TNFα (Figure 3F, *p* = 0.05), which were statistically significant only in the case of CCL2 [two-way ANOVA F_infection_(1, 10) = 5.89, *p* = 0.03], IL-6 [two-way ANOVA F_infection_ (1, 10) = 5.57, *p* = 0.03], and TNFα [two-way ANOVA F_infection_ (1, 10) = 7.71, *p* = 0.02]. Interestingly, H7N7 infection did not significantly increase the above chemokines and cytokines in the brains of previously vaccinated mice compared with vaccinated mice receiving PBS (CCL2: *p* = 0.44, IFNγ: *p* = 0.87, IL1β: *p* = 0.68, IL-6: *p* = 0.18, TNFα: *p* = 0.14). In the young mice, the levels of IL-10 (Figure 3E) did not change significantly in the brains of the two groups infected with H7N7, regardless of whether they were vaccinated or not [two-way ANOVA F_infection_ (1, 10) = 0.63, *p* = 0.44].

As in the lungs, a comparison between the two groups receiving PBS (Ctrl-PBS and VAC-PBS) in the young mice showed that vaccination *per se* did not significantly alter the levels of inflammatory mediators, at least at this time point, which was 22 days after the second immune boost (Figures 3A–F).

Analysis of cytokine and chemokine levels in the brains of aged mice revealed a somewhat different picture than in young mice (Figures 3G–L).

Infection with the H7N7 resulted in increased levels of CCL2 (Figure 3G), IFNγ (Figure 3H), IL1β (Figure 3I), IL-6 (Figure 3J), and TNFα (Figure 3L) in the brains of mice, whether or not they had been previously vaccinated. This increase was statistically significant in the case of CCL2 [two-way ANOVA F_infection_(1, 8) = 6.64, *p* = 0.03] and IL1β [two-way ANOVA F_infection_(1, 8) = 6.88, *p* = 0.03]. Interestingly, even the levels of CCL2 (Figure 3G, *p* = 0.01) and IFNγ (Figure 3H, *p* = 0.02) were higher in the brains of infected mice that had been previously vaccinated than in unvaccinated infected mice.

In contrast to young mice, infection with the H7N7 in the older mice resulted in increased levels of IL-10 in the brains of the unvaccinated mice [Figure 3K, two-way ANOVA F_infection_(1, 8) = 11.40, *p* = 0.009] compared with the corresponding control mice (Ctrl-PBS, *p* = 0.01). However, this increase was not observed in the brains of H7N7 infected mice that had been previously vaccinated compared with vaccinated mice that received PBS (*p* = 0.10).

Overall, these results suggest that infection with the H7N7 increases the levels of inflammatory mediators in the lungs and brains of young and old mice. Vaccination indeed largely prevented this increase in the lungs and brains of young mice, which was not the case in older animals. Surprisingly, even vaccination in the older mice resulted in an excessive release of certain cytokines and chemokines triggered by infection, illustrating the altered immune response in aging.

### 3.3 Effect of immunization on neuroinflammation induced by H7N7 infection

Inflammation is triggered by pathogens such as viruses and leads to various biochemical cascades aimed at eliminating these threats to homeostasis. In the brain, however, neuroinflammation is manifested by elevated levels of proinflammatory cytokines, increased numbers and activation status of microglia, and peripheral leukocytes infiltration that can lead to neuronal damage (Woodburn et al., 2021). Previously, 7 days after infection with PR8 H1N1 IAV, an increased proportional area of IBA-1^+^ microglia in the CA1, CA3, and *dentate gyrus* subregions of the hippocampus, a brain region susceptible to neuroinflammation was documented (Jurgens et al., 2012). In addition, several studies have shown that the density of microglia in the various subregions of the hippocampus and even in other brain regions such as the *substantia nigra pars compacta* increased well beyond the acute phase of H7N7 (Hosseini et al., 2018), CA/09 H1N1 (Sadasivan et al., 2015), and H5N1 IAV infection (Jang et al., 2012).

**FIGURE 4:**
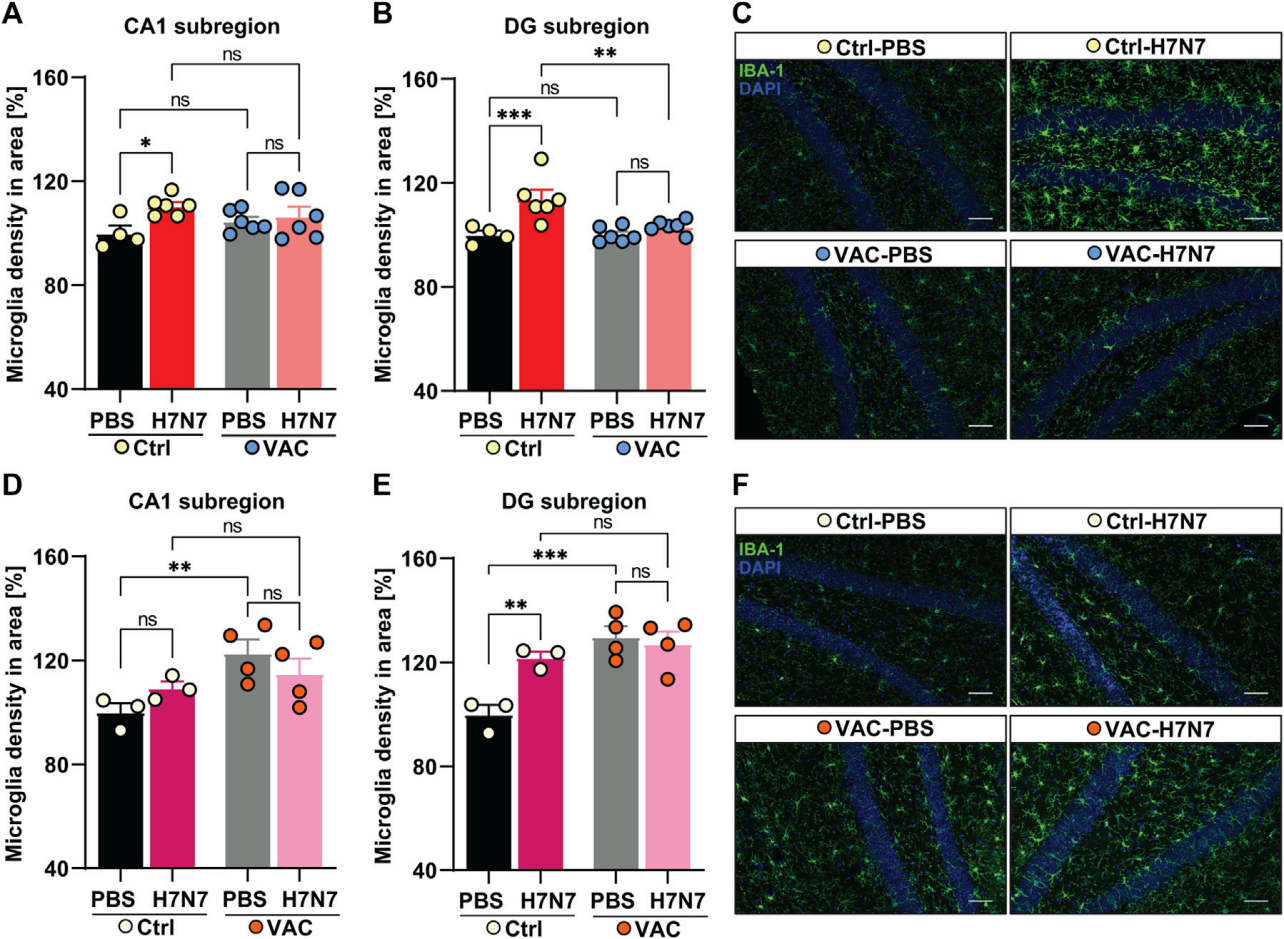
Effects of vaccination on hippocampal neuroinflammation induced by H7N7 IAV infection. In young mice **(A)** microglial density in the CA1 subregion of the hippocampus was significantly increased 8 days after H7N7 IAV infection in unvaccinated mice compared with the corresponding control. In vaccinated mice, this increase was not present after H7N7 IAV infection. Vaccination itself did not result in the significant increase in microglial density in CA1 (*n* = 4–6 animals per group). **(B)** Infection with the H7N7 IAV resulted in a significant increase in microglial density in the dentate gyrus subregion in unvaccinated mice compared with the control group. This increase was not seen after H7N7 IAV infection in previously vaccinated mice. Vaccination itself did not lead to the significant increase in microglial density in DG (*n* = 4–6 animals per group). **(C)** Representative examples of IBA-1 immunostaining in the *dentate gyrus* subregion at 8 dpi (20X); scale = 50 μm. In aged mice **(D)** 10 days after H7N7 IAV infection, microglia density in the CA1 subregion of the hippocampus was comparable between unvaccinated infected and PBS-inoculated mice. Microglia density was significantly increased in vaccinated mice receiving PBS compared with unvaccinated mice receiving PBS. However, H7N7 IAV infection did not further increase microglial cell density in vaccinated mice (*n* = 3–4 animals per group). **(E)** In the DG subregion, H7N7 IAV infection in unvaccinated mice resulted in increased microglia density compared with corresponding control mice. Microglial density was also significantly increased in this subregion of the hippocampus in vaccinated mice receiving PBS compared with unvaccinated mice receiving PBS. However, infection with H7N7 IAV did not further increase microglial cell density in vaccinated mice (*n* = 3–4 animals per group). **(F)** Representative examples of IBA-1 immunostaining in the *dentate gyrus* subregion at 10 dpi (20X); scale = 50 μm. Data are presented as mean ± SEM and were analyzed with an ordinary two-way ANOVA followed by Fisher’s LSD test; *p < 0.05, **p < 0.01, and **p < 0.01.

To determine whether vaccination is able to prevent the neuroinflammation induced by H7N7, the density of microglia in the CA1 and *dentate gyrus* subregions of the hippocampus was examined in all experimental groups (Figure 4).

In 2-month-old mice, 8 days post infection with H7N7 resulted in a significant increase in microglial cell density in the CA1 (Δ10%, *p* = 0.01, Figure 4A) and *dentate gyrus* (Δ13%, *p* = 0.0005, Figures 4B, C) hippocampal subregions of unvaccinated mice compared with control mice inoculated with PBS, confirming neuroinflammation induced by H7N7 during the acute phase of the disease [CA1: two-way ANOVA F_infection_(1, 18) = 5.74, *p* = 0.02; DG: two-way ANOVA F_infection_(1, 18) = 14.98, *p* = 0.001]. However, examination of the vaccinated mice showed that there were no significant differences in microglial density in either subregion of the hippocampus (CA1: Δ2%, *p* = 0.57; DG: Δ3%, *p* = 0.29) between the mice infected with H7N7 that had been previously vaccinated and the respective control animals (Figures 4A–C). In the *dentate gyrus* subregion, mice infected with H7N7 that had been previously vaccinated had significantly lower numbers of microglial cells (Δ10%, *p* = 0.001) compared with unvaccinated infected mice [two-way ANOVA F_vaccination_(1, 18) = 5.79, *p* = 0.02]. Although vaccination *per se* did not appear to significantly alter hippocampal microglial density, the number of microglia in the CA1 subregion was slightly higher (Δ5%, *p* = 0.25, Figure 4A) in vaccinated mice receiving PBS compared with unvaccinated mice inoculated with PBS.

In 15-month-old mice (Figures 4D–F), the results differed from young animals. Analysis of microglia density in hippocampal subregions at day 10 post-infection showed that although H7N7 infection resulted in an non-significant increase in the number of microglia in CA1 subregion (Δ9%, *p* = 0.25, Figure 4D) in unvaccinated mice compared with PBS inoculated mice, however, the density of microglia was significantly increased in *dentate gyrus* subregion (Δ20%, *p* = 0.005, Figures 4E, F) again confirming the neuroinflammation triggered by H7N7 infection [CA1: two-way ANOVA F_infection_(1, 10) = 0.01, *p* = 0.89; DG: two-way ANOVA F_infection_(1, 10) = 5.29, *p* = 0.04].

In aged mice, comparison between two groups receiving PBS (Figures 4D, E) showed that vaccination *per se* resulted in an significant increased numbers of microglial cells in both subregions of the hippocampus [CA1: Δ20%, *p* = 0.009, two-way ANOVA F_vaccination_(1, 10) = 7.78, *p* = 0.01; DG: Δ25%, *p* = 0.0005, two-way ANOVA F_vaccination_(1, 10) = 17.66, *p* = 0.001]. However, no further significant increase was detectable in the H7N7 infected mice that had been previously vaccinated compared with vaccinated mice receiving PBS (CA1: Δ6%, *p* = 0.26; DG: Δ2%, *p* = 0.62).

Overall, these results showed that vaccination plays a preventive role against neuroinflammation induced by H7N7 infection in young mice. However, this effect was not very clear in older mice, and apparently vaccination itself appears to induce proliferation of microglia in the hippocampus of older mice.

In addition to increased numbers of microglial cells as a hallmark of neuroinflammation, one of the common metrics used to assess microglial activation is a broad morphological change from a small soma with branched processes in homeostasis to an amoeboid with thicker and less complex processes during neuroinflammation (Woodburn et al., 2021). Indeed, initial reports indicated that viral infections, including IAV infection, cause microglial activation based mainly on changes in morphological features (Hosseini et al., 2018; da Silva Creão et al., 2021). The main phenotypic changes in activated microglia are an increase in soma volume, retraction of processes, and loss of uniform tissue distribution (Arcuri et al., 2017).

**FIGURE 5:**
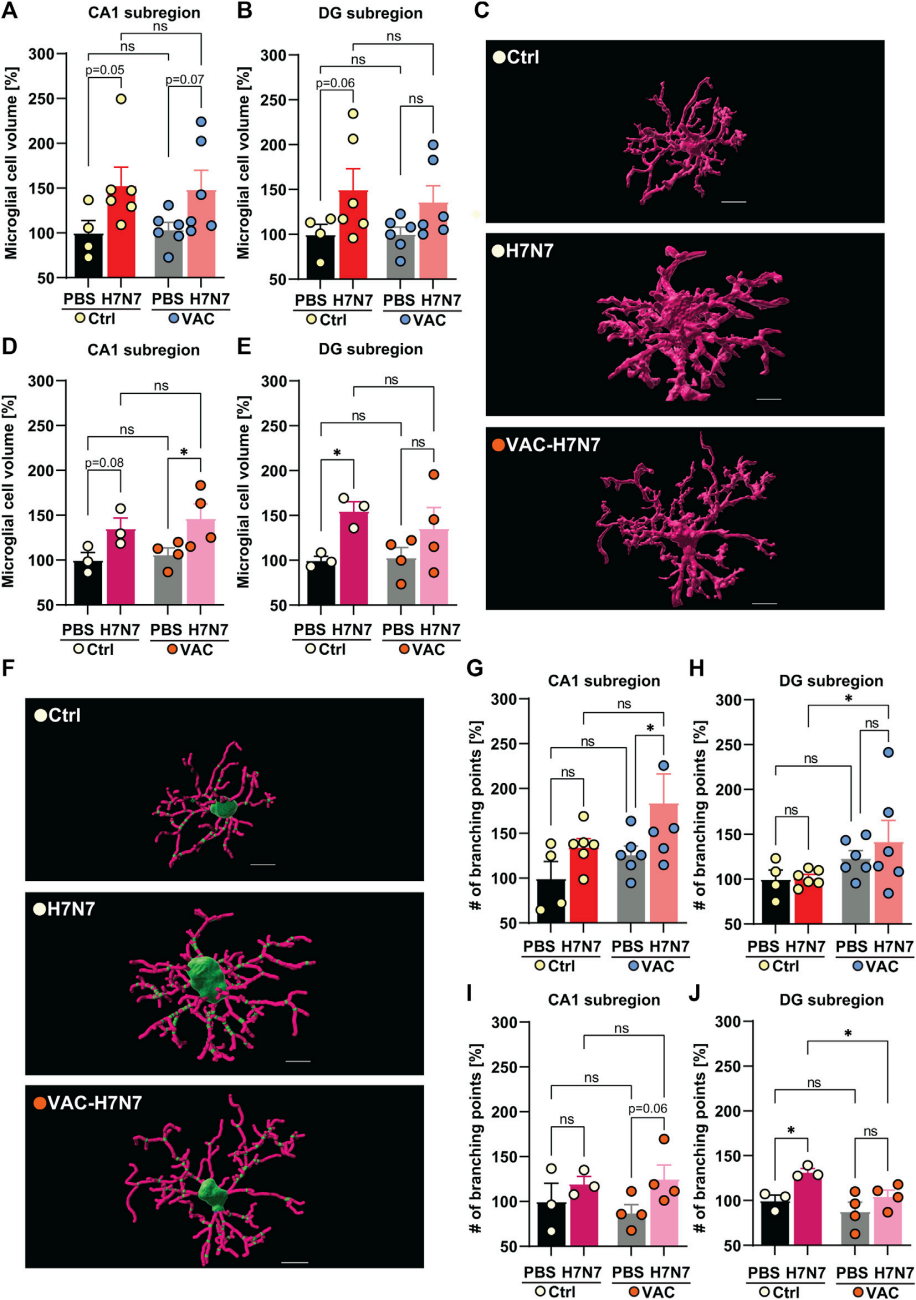
Effects of vaccination on microglial morphological changes induced by H7N7 IAV infection. In young mice **(A)** microglial cell volume in the CA1 subregion of the hippocampus was increased 8 days after infection with the H7N7 IAV in both unvaccinated and vaccinated mice compared with the corresponding control groups receiving PBS (*n* = 4–6 animals per group). **(B)** Infection with the H7N7 IAV resulted in an increase in the volume of microglial cells in the *dentate gyrus* subregion in unvaccinated mice compared with the control group. This increase was seen to a lesser extent after infection with the H7N7 IAV in previously vaccinated mice. Vaccination itself did not significantly increase microglial cell volume in either subregion (*n* = 4–6 animals per group). **(C)** Representative 3D reconstruction of a microglial cell volume in the *dentate gyrus* subregion of unvaccinated and vaccinated older animals after infection compared with PBS inoculation; scale = 7 μm. In aged mice, at 10 days after H7N7 IAV infection, microglial cell volume increased in **(D)** CA1 and **(E)** DG subregions compared with unvaccinated PBS-inoculated mice. Microglial cell volume was significantly increased in the CA1 subregion of vaccinated mice infected with H7N7 IAV compared with vaccinated mice receiving PBS. Vaccination itself did not significantly alter microglial cell volume in either subregion (*n* = 3–4 animals per group). **(F)** Representative 3D reconstruction of soma (green), filaments (pink), and branch points (small green dots in filaments) in the *dentate gyrus* subregion of unvaccinated and vaccinated older animals after infection compared with PBS inoculation; scale = 7 μm. In young mice, **(G)** the number of branch points of microglial processes in the CA1 subregion increased after H7N7 IAV infection in previously vaccinated mice compared with vaccinated mice receiving PBS. **(H)** In the DG subregion, infection induced a higher number of branch points in microglial processes in previously vaccinated mice compared with unvaccinated, infected mice (*n* = 4–6 animals per group). In aged mice, **(I)** H7N7 IAV infection resulted in a substantial increase in branch points of microglial processes in the CA1 subregion in previously vaccinated mice compared with vaccinated mice receiving PBS. **(J)** In the DG subregion, infection resulted in a significantly increased number of branch points of microglial processes in the unvaccinated mice compared with the corresponding control. However, in the vaccinated mice, infection resulted in a decreased number of branch points of microglial processes compared with the unvaccinated infected mice (*n* = 3–4 animals per group). Vaccination itself did not significantly alter the number of branch points of microglial processes in either subregion of the hippocampus, in either the young or old cohorts. Data are presented as mean ± SEM and were analyzed with an ordinary two-way ANOVA followed by Fisher’s LSD test; *p < 0.05.

Therefore, the next step after IMARIS 3D reconstruction was to analyze the total volume and number of branching points of randomly selected microglial cells in the CA1 and *dentate gyrus* subregions of the hippocampus in all experimental groups (Figure 5).

In the young cohort, H7N7 infection resulted in an increase in total microglial cell volume in CA1 (Δ53%, *p* = 0.05, Figure 5A) and *dentate gyrus* (Δ50%, *p* = 0.06, Figures 5B, C) in the hippocampal subregions of unvaccinated mice compared to control mice receiving PBS, indicating neuroinflammation induced by H7N7 during the acute phase of the disease, although multiple comparisons between groups did not reach the significance level [CA1: two-way ANOVA F_infection_(1, 18) = 7.67, *p* = 0.01; DG: two-way ANOVA F_infection_(1, 18) = 6.21, *p* = 0.02].

In the vaccinated mice, H7N7 infection also resulted in an increase in the volume of microglial cells in the CA1 subregions compared to the vaccinated mice receiving PBS, although this increase did not reach the significance level statistically (Δ43%, *p* = 0.07, Figure 5A), but in the *dentate gyrus* subregion this increase was observed to a lesser extent (Δ35%, *p* = 0.13, Figure 5B). In both hippocampal subregions, vaccination *per se* did not appear to significantly alter microglial cell volume in vaccinated mice receiving PBS compared to unvaccinated mice inoculated with PBS (CA1: Δ4%, *p* = 0.88; DG: Δ0.5%, *p* = 0.98).

The same trends were observed in the age cohort (Figures 5D, E). In unvaccinated mice, H7N7 infection induced an increased volume of microglial cells in CA1 (Δ36%, *p* = 0.08, Figure 5D) and even more markedly in the *dentate gyrus* subregion (Δ55%, *p* = 0.04, Figure 5E) compared with control mice inoculated with PBS [CA1: two-way ANOVA F_infection_(1, 10) = 9.82, *p* = 0.01; DG: two-way ANOVA F_infection_(1, 10) = 7.50, *p* = 0.02].

However, after vaccination, the larger microglial cells were located in the CA1 subregion of the infected mice compared with vaccinated mice receiving PBS (Δ38%, *p* = 0.02, Figure 5D). As in the young mice, the increased volume of microglial cells caused by infection was also present to a lesser extent in the *dentate gyrus* region of the vaccinated mice (Δ31%, *p* = 0.15, Figure 5E). Here, a comparison of two uninfected groups receiving PBS showed that vaccination *per se* did not induce significant changes in microglial cell volume in either hippocampal subregion in aged mice (CA1: Δ7%, *p* = 0.70; DG: Δ4%, *p* = 0.88).

As previously mentioned, the reduced complexity of microglial processes is also known as one of the stages of microglial activation. However, microglial activation has been shown to represent a continuum between ramified activated and amoeboid forms (Ladeby et al., 2005). To provide a further indication of the morphological changes in microglia, the number of branch points of microglial filaments in all experimental groups was assessed here according to the IMARIS 3D reconstruction of microglial processes (Figures 5F–J).

The results in young mice showed that H7N7 infection in the CA1 subregion resulted in a significantly increased number of branch points of microglial processes in the vaccinated mice compared with the corresponding control group in which the vaccinated animals received PBS [Δ46%, *p* = 0.04; two-way ANOVA F_infection_(1, 18) = 5.17, *p* = 0.03, Figure 5G]. In the *dentate gyrus* subregion, infection also apparently induced more complex microglial processes in the previously vaccinated mice compared with the unvaccinated infected mice [Δ40%, *p* = 0.04; two-way ANOVA F_vaccination_(1, 18) = 4.97, *p* = 0.03, Figure 5H]. However, in both subregions, H7N7 infection did not significantly alter the number of branch points of microglial processes in the unvaccinated mice, although the CA1 subregion was more affected (CA1: Δ35%, *p* = 0.26; DG: Δ2%, *p* = 0.94). Comparison of the two groups receiving PBS also revealed no significant changes in the complexity of microglial processes (CA1: Δ26%, *p* = 0.39; DG: Δ23%, *p* = 0.28).

In the CA1 subregion of aged mice (Figure 5I), infection with H7N7 in previously vaccinated mice resulted in an increase in the branch points of microglial processes compared with vaccinated mice receiving PBS, although this change was not statistically significant [Δ43%, *p* = 0.06; two-way ANOVA F_infection_(1, 10) = 4.31, *p* = 0.06, Figure 5I].

The picture of microglial branching complexity in the *dentate gyrus* subregion (Figure 5J) was somewhat different in the older animals than in the young mice. H7N7 infection in unvaccinated mice resulted in significantly increased complex branching of microglial cells compared to unvaccinated mice receiving PBS [Δ32%, *p* = 0.02; two-way ANOVA F_infection_(1, 10) = 9.97, *p* = 0.01, Figure 5J].

Of note, in the *dentate gyrus* subregion of the older group, infection with H7N7 did not significantly alter microglial complexity in the previously vaccinated mice compared with the vaccinated mice receiving PBS (Δ19%, *p* = 0.12, Figure 5J), but infection resulted in significantly fewer branch points of microglial processes in the vaccinated mice than in the unvaccinated infected mice [Δ21%, *p* = 0.03; two-way ANOVA F_vaccination_(1, 10) = 6.33, *p* = 0.03, Figure 5J]. Again, no significant differences were observed between the two PBS-inoculated group (CA1: Δ12%, *p* = 0.54; DG: Δ11%, *p* = 0.30).

Taken together, these results showed changes in microglial cell morphological characteristics after H7N7 infection that can be modulated if mice were vaccinated before infection. Moreover, this modulation was different in young versus old animals.

### 3.4 Effect of immunization against hippocampal neuron morphology alterations induced by H7N7 IAV infection

**FIGURE 6:**
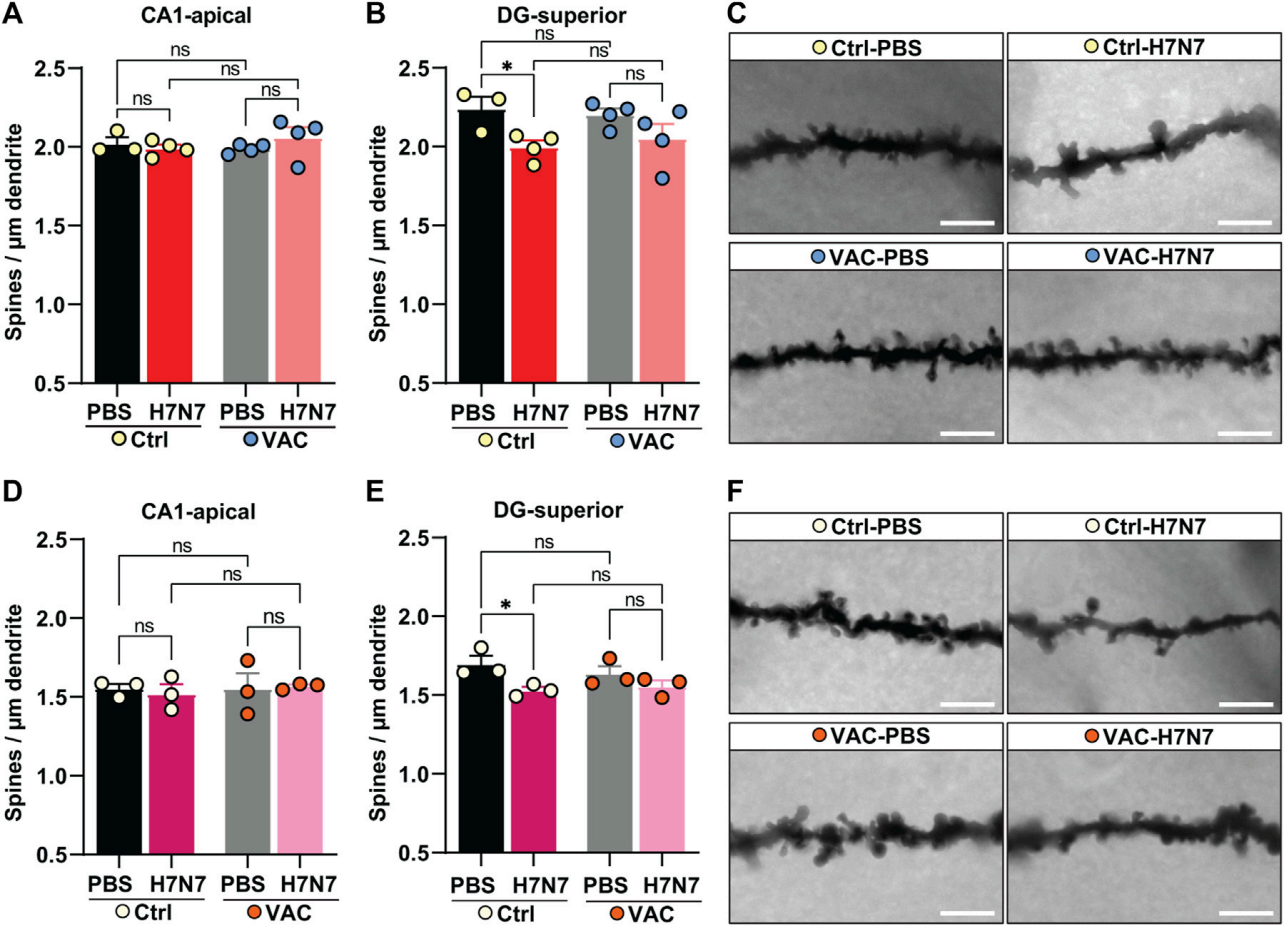
Effects of vaccination on hippocampal neuron morphology changes caused by H7N7 IAV infection. In young mice **(A)** the density of dendritic spines in the CA1 subregion of the hippocampus did not change significantly 8 days after infection with the H7N7 IAV in unvaccinated mice compared with the corresponding control group. This was also the case in vaccinated mice. Vaccination itself did not cause the significant changes in dendritic spine density in CA1. **(B)** Infection with the H7N7 IAV resulted in a significant reduction in dendritic spine density in the *dentate gyrus* subregion in unvaccinated mice compared with the control group. This significant reduction was not observed after H7N7 IAV infection in previously vaccinated mice. Vaccination itself did not induce the significant changes in dendritic spine density in DG (*n* = 3–4 animals per group). **(C)** Representative images of Golgi-Cox staining showing dendrites of granule cells in the *dentate gyrus superior* subregion of all experimental groups of young mice (63X); scale = 5 μm. In aged mice **(D)**, the dendritic spine density in the CA1 subregion of the hippocampus did not change significantly 10 days after infection with the H7N7 IAV in unvaccinated mice compared to the corresponding control group. This was also the case in vaccinated mice. Vaccination itself did not cause the significant changes in dendritic spine density in CA1. **(E)** Infection with the H7N7 IAV resulted in a significant reduction in dendritic spine density in the superior subregion of the *dentate gyrus* in unvaccinated mice compared to controls. This significant reduction was not observed after H7N7 IAV challenge in previously vaccinated mice. Vaccination itself did not lead to significant changes in dendritic spine density in DG (*n* = 3 animals per group). **(F)** Representative Golgi-Cox staining images showing dendrites of granule cells in the *superior dentate gyrus* subregion of all experimental groups of old mice (63X); Scale = 5 µm. Data are presented as mean ± SEM and were analyzed with an ordinary two-way ANOVA followed by Fisher’s LSD test; *p < 0.05.

Prominent changes in microglial density and activation status observed here may be associated with functional changes in these cells. As the brain-resident macrophages, microglia are capable of removing pathogens, aberrant synaptic connections between neurons, and other cellular debris under pathological conditions (Boche et al., 2013). Therefore, the next step was to analyze dendritic spine density as a morphological indicator of hippocampal neurons in all experimental groups. Spines are tiny dendritic protrusions that carry the majority of excitatory synapses in the hippocampus. Changes in spine density may provide information about changes in connectivity of hippocampal subregions. Therefore, spines were counted separately on the apical dendrites of CA1 pyramidal neurons and on granule cells in the superior leaflet of the *dentate gyrus* in the hippocampus (Figure 6).

In both young and old mice (Figures 6A, D), H7N7 infection during the acute phase of disease did not result in the loss of spines in the apical dendrites of CA1 neurons, whether or not the mice had been previously vaccinated [young: two-way ANOVA F_infection_(1, 11) = 0.22, *p* = 0.64, Figure 6A; old: two-way ANOVA F_infection_(1, 8) = 0.02, *p* = 0.88, Figure 6D]. A comparison of the two control groups receiving PBS showed that vaccination *per se* did not induce significant changes in dendritic spine density in this subregion in either cohort [young: two-way ANOVA F_vaccination_(1, 11) = 0.17, *p* = 0.68, Figure 6A; two-way ANOVA F_vaccination_(1, 8) = 0.15, *p* = 0.70, Figure 6D].

Analysis of spine density in the dendrites of granule cells located in the superior leaflet of the d*entate gyrus* showed that infection with H7N7 resulted in significantly decreased dendritic spine density in unvaccinated young [Δ11%, *p* = 0.02; two-way ANOVA F_infection_(1, 11) = 8.95, *p* = 0.01, Figures 6B,C] and aged mice [Δ10%, *p* = 0.01; two-way ANOVA F_infection_(1, 8) = 9.23, *p* = 0.01, Figures 6E,F] compared with matched controls receiving PBS. Remarkably, H7N7 infection in both young (Δ6%, *p* = 0.12, Figures 6B,C) and old (Δ4%, *p* = 0.20, Figures 6E,F) vaccinated mice did not result in significant loss of dendritic spines in the *dentate gyrus* subregion compared with vaccinated mice receiving PBS. Comparison of the two control groups receiving PBS in both cohorts showed that although vaccination *per se* resulted in a slight decrease in dendritic spine density in the *dentate gyrus* subregion, this change was not statistically significant (young: Δ2%, *p* = 0.69; old: Δ3%, *p* = 0.30).

Thus, these findings showed that vaccination is able to prevent dendritic spine loss caused by H7N7 infection in both young and old mice.

### 3.5 The role of microglia in synaptic remodeling induced by H7N7 infection during the acute phase of the disease

**FIGURE 7:**
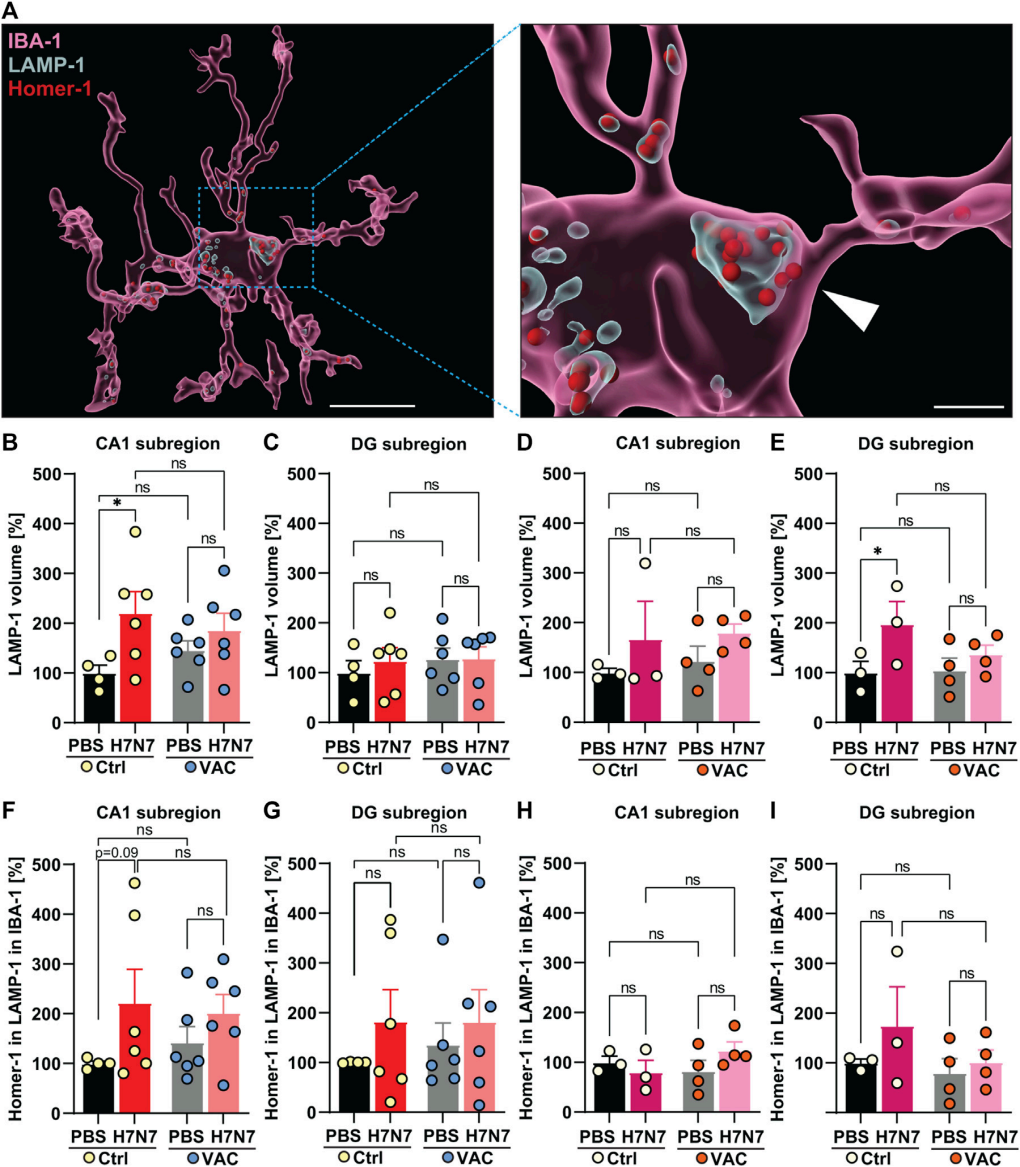
Synaptic engulfment by microglia induced by H7N7 IAV infection and its possible modulation by vaccination. **(A)** Representative reconstruction of a microglial cell, a lysosomal compartment, and Homer-1 positive postsynaptic terminals from the *dentate gyrus* hippocampal subregion of aged mice. The entire microglial cell is shown on the left image (Scale = 10 µm), while an enlarged section is shown on the right side. IBA-1 surface was remodeled in IMARIS, as well as LAMP-1 volume inside the IBA-1 and Homer-1 puncta, which were localized inside the LAMP-1 volume inside the IBA-1 surface (Scale = 2 µm). In young mice **(B)**, the LAMP-1 labeled lysosome volume in the microglial cells in the CA1 subregion of the hippocampus was significantly increased 8 days after infection with the H7N7 IAV in unvaccinated mice compared to the corresponding control group. This increase was less pronounced in vaccinated mice. In the CA1 subregion, vaccination itself did not cause significant changes in lysosome volume within the microglial cells of either PBS-inoculated group. **(C)** No significant changes were observed in the *dentate gyrus* of young mice after H7N7-IAV challenge, either between unvaccinated mice or between vaccinated mice. Vaccination *per se* did not also cause significant differences in this subregion (*n* = 4–6 animals per group). In aged mice, **(D)** in the CA1 subregion, infection resulted in increased amounts of lysosome volume regardless of whether the mice had been previously vaccinated, although the difference was not statistically significant. **(E)** In the DG subregion, the volume of lysosomes in microglial cells increased in unvaccinated infected mice, which was not very evident in vaccinated mice. The vaccination itself did not lead to any significant changes (*n* = 3 animals per group). **(F,G)** In both hippocampal subregions of young mice, H7N7 IAV infection resulted in increased Homer 1-positive puncta engulfment by microglial cells in unvaccinated mice compared to matched control mice. However, this trend of increase was less pronounced in vaccinated infected mice than in vaccinated mice receiving PBS. Vaccination *per se* did not lead to any significant trends (*n* = 4–6 animals per group). In older mice, **(H)** Homer-1 puncta engulfment by microglial cells in the CA1 subregion did not change in all experimental groups, **(I)** but in the DG subregion, the number of Homer-1 puncta engulfment by microglial cells showed stronger increasing trends that were absent in vaccinated infected mice. Even in aged mice, vaccination *per se* did not lead to any significant trends in either subregion. Data are presented as mean ± SEM and were analyzed with an ordinary two-way ANOVA followed by Fisher’s LSD test; *p < 0.05.

Exaggerated inflammatory responses by activated microglia, as demonstrated here, may impair homeostatic functions and lead to neuronal damage, including impairment of synaptic connectivity (Lima et al., 2022). Therefore, neuronal damage triggered by H7N7 IAV could be mediated by microglia that engulf and digest synaptic terminals by phagocytosis with the help of lysosomes. As dynamic organelles of cell catabolism, the number, size, and distribution of lysosomes can adapt to the individual needs of a cell at a given time point (de Araujo et al., 2020). Since most phagocytosed components are expected to be degraded in lysosomal vesicles, in the next step it was assumed that the total volume of lysosomes in microglia is proportional to its phagocytic activity. Thus, volumetric changes in microglial lysosomes were examined in all experimental groups (Figure 7A).

In 2-month-old mice, H7N7 at day 8 post-infection resulted in a significant increase in lysosome volume labeled with LAMP-1 in microglial cells in the CA1 subregion of the hippocampus [Δ121%, *p* = 0.02, two-way ANOVA F_infection_(1, 18) = 6.05, *p* = 0.02, Figure 7B] of unvaccinated mice compared to control mice inoculated with PBS. In contrast to this, examination of the vaccinated mice showed that there were no significant differences in lysosome volume in this subregion (Δ28%, *p* = 0.36) between the mice infected with H7N7 that had been previously vaccinated and the vaccinated mice that received PBS (Figure 7B). It is important to note that vaccination itself resulted in a considerable increase in lysosome volume in the CA1 subregion compared between unvaccinated and vaccinated mice receiving PBS (Δ46%, *p* = 0.35). However, in the *dentate gyrus* subregion (Figure 7C), infection apparently did not result in a significant further increase in lysosome volume after H7N7 infection when comparing both unvaccinated (Δ24%, *p* = 0.53) or vaccinated mice [Δ1%, *p* = 0.97, two-way ANOVA F_infection_(1, 18) = 0.24, *p* = 0.62, Figure 7C]. Although the difference in lysosome volume in this subregion was also less pronounced in infected mice that had been previously vaccinated, lysosome volume was generally higher than in the CA1 subregion at baseline, but values are normalized to the Ctrl-PBS group for consistency between all graphs.

In 15-month-old mice (Figures 7D, E), the results were again different from those in young mice. Analysis of lysosome volume in microglial cells in hippocampal subregions at day 10 post-infection showed that although infection with H7N7 resulted in a non-significant increase in lysosome volume in microglia in the CA1 subregion (Δ67%, *p* = 0.27, Figure 7D) in unvaccinated mice compared with PBS-inoculated mice. This increase was also observed after infection of vaccinated mice (Δ46%, *p* = 0.28) compared with vaccinated mice receiving PBS [two-way ANOVA F_infection_(1, 10) = 2.63, *p* = 0.13, Figure 7D]. Again, in the *dentate gyrus* subregion (Figure 7E), although the baseline level of lysosomes in microglial cells was higher than in the CA1 subregion, infection resulted in a significant increase in the volume of lysosomes in microglia in this subregion [Δ98%, *p* = 0.04, two-way ANOVA F_infection_(1, 10) = 5.36, *p* = 0.04, Figure 7E]. In aged mice, vaccination *per se* did not result in significant differences between the two PBS-inoculated groups in the both subregions of the hippocampus (CA1: Δ23%, *p* = 0.67; DG: Δ4%, *p* = 0.91).

Given the phagocytic appearance of microglial cells in the hippocampal subregions of both the young and old cohorts, as determined by morphological and lysosomal analyzes, the hypothesis of whether phagocytic microglial cells are capable of promoting synapse loss in H7N7 IAV infection was next examined (Figure 7A). To this end, the number of Homer-1-positive puncta in microglial cells was analyzed in all experimental groups. Homer-1 is a synaptic scaffold protein in postsynaptic terminals that regulates glutamatergic synapses and spine morphogenesis (Yoon et al., 2021).

In the young cohort, increased numbers of Homer-1 puncta in lysosomes were detected in microglial cells of unvaccinated H7N7 IAV-infected mice compared with control mice inoculated with PBS in both subregions of the hippocampus [CA1: Δ122%, *p* = 0.09, two-way ANOVA F_infection_(1, 18) = 3.79, *p* = 0.06; DG: Δ83%, *p* = 0.34, two-way ANOVA F_infection_(1, 18) = 1.28, *p* = 0.27, Figures 7F, G].

This increase was less pronounced in infected mice that had been previously vaccinated compared to the corresponding vaccinated mice that received PBS (CA1: Δ43%, *p* = 0.34; DG: Δ34%, *p* = 0.55, Figures 7F, G). When comparing the two groups that received PBS, although the vaccination itself resulted in a change in Homer-1-positive puncta engulfed by microglia in both hippocampal subregions, these changes were not statistically significant (CA1: Δ42%, *p* = 0.55; DG: Δ36%, *p* = 0.67).

In the older mice, there were no significant differences in the number of engulfed Homer-1-positive terminals in the lysosomes of microglial cells induced by H7N7 IAV infection in unvaccinated mice (Δ20%, *p* = 0.52, Figure 7H) and vaccinated mice (Δ50%, *p* = 0.14, Figure 7H) compared with the corresponding control groups receiving PBS in the CA1 subregion, although this postsynaptic engulfment induced by the infection was apparently more pronounced in older mice even after vaccination [two-way ANOVA F_infection_(1, 10) = 0.29, *p* = 0.59, Figure 7H].

Remarkably, H7N7 IAV infection in the *dentate gyrus* subregion of aged unvaccinated mice resulted in an increase in the number of postsynaptic terminals engulfed by microglial cells (Δ75%, *p* = 0.24, Figure 7I), which was not observed in infected mice that had been previously vaccinated (Δ28%, *p* = 0.68, Figure 7I) compared with the corresponding control groups that received PBS, although all these changes were not statistically significant [two-way ANOVA F_infection_(1, 10) = 1.46, *p* = 0.25, Figure 7I]. In aged mice, comparison of the two PBS-inoculated groups showed that vaccination *per se* did not appear to affect microglia-mediated postsynaptic engulfment in either hippocampal subregion [CA1: Δ18%, *p* = 0.53; DG: Δ20%, *p* = 0.72, Figures 7H, I).

Taken together, these results provided evidence that infection with H7N7 promotes phagocytic activity and engulfment of synaptic compartments by microglia in different subregions of the hippocampus, which is modulated by vaccination.

### 3.6 Effect of immunization on astrocyte reactivity triggered by H7N7 infection

In addition to microglia, astrocytes are also important regulators of innate and adaptive immune responses in the CNS. Depending on the timing and context, astrocyte activity can exacerbate inflammatory responses and tissue damage or promote immunosuppression and tissue repair. To initially assess astrocyte reactivity, GFAP staining of brain tissue from all experimental groups was performed. Previously, it was shown that reactive astrocytes exhibit a striking increase in GFAP immunoreactivity (astrogliosis) (Wilhelmsson et al., 2006). Therefore, the fluorescent intensity of GFAP staining was examined here as a marker of astrocytic reactivity in the CA1 and DG subregions of the hippocampus.

**FIGURE 8:**
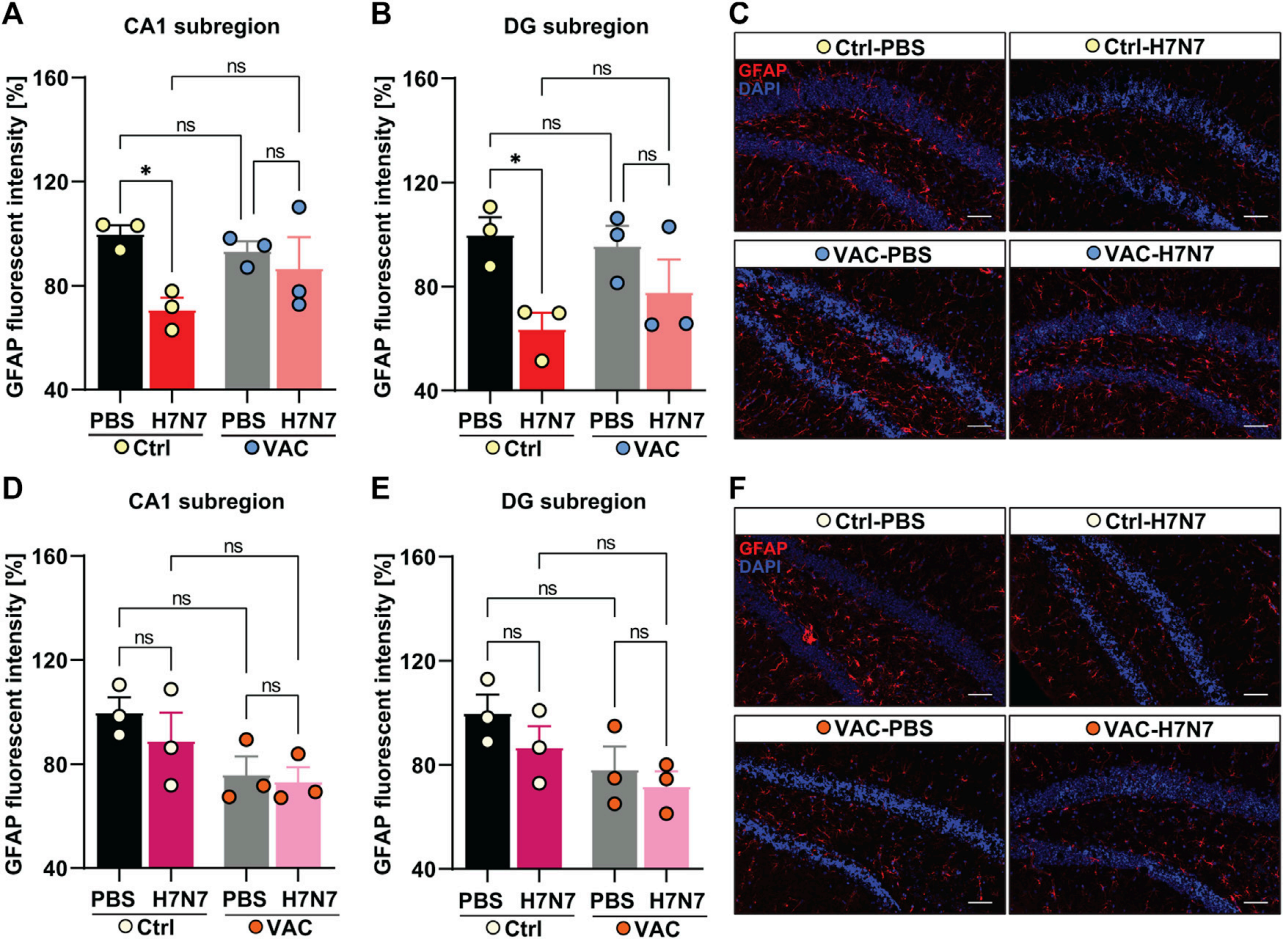
Effects of vaccination on hippocampal GFAP expression levels induced by H7N7 IAV infection. In young mice **(A)** GFAP expression in the CA1 subregion of the hippocampus was significantly decreased 8 days after H7N7 IAV infection in unvaccinated mice compared with the corresponding control. This decrease was not present in vaccinated mice after H7N7 IAV infection. Vaccination itself did not significantly decrease GFAP expression levels in CA1 (*n* = 3 animals per group). **(B)** Infection with H7N7 IAV resulted in a significant decrease in GFAP expression levels in the *dentate gyrus* subregion in unvaccinated mice compared with the control group. This decrease was not observed after infection with the H7N7 IAV in previously vaccinated mice. Vaccination itself did not induce the significant changes in DG (*n* = 3 animals per group). **(C)** Representative examples of GFAP immunostaining in the *dentate gyrus* subregion at 8 dpi (20X); scale = 50 μm. In aged mice **(D)**, 10 days after H7N7 IAV infection, GFAP expression in the CA1 subregion of the hippocampus was comparable between unvaccinated infected and PBS-vaccinated mice. GFAP expression was lower in vaccinated mice receiving PBS than in unvaccinated mice receiving PBS. However, infection with H7N7 IAV did not further decrease GFAP expression in vaccinated mice (*n* = 3 animals per group). **(E)** In the DG subregion, H7N7 IAV infection in unvaccinated mice resulted in slightly decreased GFAP expression compared to corresponding control mice. GFAP expression was also reduced in this subregion of the hippocampus in vaccinated mice receiving PBS compared with unvaccinated mice receiving PBS. However, infection with H7N7 IAV did not further reduce GFAP expression in vaccinated mice (*n* = 3 animals per group). **(F)** Representative examples of GFAP immunostaining in the *dentate gyrus* subregion at 10 dpi (20X); scale = 50 μm. Data are presented as mean ± SEM and were analyzed with an ordinary two-way ANOVA followed by Fisher’s LSD test; *p < 0.05, **p < 0.01, and **p < 0.01.

In 2-month-old mice, 8 days post infection with H7N7 a significant decrease in GFAP fluorescent intensity was evident in the CA1 (Δ30%, *p* = 0.01, Figure 8A) and *dentate gyrus* (Δ36%, *p* = 0.01, Figures 8B, C) hippocampal subregions of unvaccinated mice compared with control mice inoculated with PBS, indicating reduced GFAP expression induced by H7N7 during the acute phase of the disease [CA1: two-way ANOVA F_infection_(1, 8) = 7.17, *p* = 0.02; DG: two-way ANOVA F_infection_(1, 8) = 9.99, *p* = 0.01]. However, examination of the vaccinated mice showed that there were no significant differences in GFAP expression levels in either subregion of the hippocampus (CA1: Δ7%, *p* = 0.50; DG: Δ20%, *p* = 0.17) between the H7N7-infected mice that had been previously vaccinated and the corresponding control animals (Figures 8A–C). In both hippocampal subregions, mice infected with H7N7 that had been previously vaccinated showed slightly higher GFAP expression than unvaccinated infected mice, but this was not statistically significant. In addition, vaccination *per se* did not appear to significantly alter GFAP expression levels in vaccinated mice receiving PBS compared with unvaccinated mice inoculated with PBS.

In 15-month-old mice (Figures 8D–F), the results differed from those of young animals. Analysis of GFAP fluorescent intensity in hippocampal subregions at day 10 post-infection showed that H7N7 infection resulted in a non-significant decrease in GFAP expression levels in the CA1 (Δ11%, *p* = 0.32, Figure 4D) and *dentate gyrus* subregion (Δ13%, *p* = 0.24, Figures 8E, F) of unvaccinated mice compared to PBS inoculated mice. In aged mice, comparison between two groups receiving PBS (Figures 8D, E) showed that vaccination *per se* resulted in a slight decrease in GFAP expression in both hippocampal subregions [CA1: Δ23%, *p* = 0.059, two-way ANOVA F_vaccination_(1, 8) = 7.03, *p* = 0.02; DG: Δ21%, *p* = 0.07, two-way ANOVA F_vaccination_(1, 8) = 6.01, *p* = 0.03]. However, there was no further significant decrease in H7N7-infected mice previously vaccinated compared to vaccinated mice receiving PBS (CA1: Δ4%, *p* = 0.80; DG: Δ8%, *p* = 0.56).

Overall, these results showed that astrocytes may also play an important inflammatory role during H7N7 IAV infection that can be modulated by immunization, particularly in young mice. However, this effect was not very clear in older mice, and apparently vaccination itself seems to induce changes in astrocytes in the hippocampus of older mice.

## 4 Discussion

Although influenza A virus is notorious for its respiratory symptoms, the most common non-respiratory complications are neurological symptoms that usually occur in the acute phase of the disease, but can also lead to long-term consequences (Ekstrand, 2012; Ludlow et al., 2016; Barbosa-Silva et al., 2018). Some IAV strains are neurotropic, such as the highly pathogenic H5N1 as well as the H7N7 IAV used in this study. These viruses are capable of invading the CNS and replicating in brain cells. IAV infection mouse models, particularly with these neurotropic viruses, have demonstrated acute and even chronic neuroinflammation with subsequent neuronal damage in several brain regions, including the hippocampus (Jang et al., 2009; Jang et al., 2012; Hosseini et al., 2018; Hosseini et al., 2021a; Hosseini and Korte, 2023), which is rich in cytokine receptors and particularly sensitive to neuroinflammation (Chesnokova et al., 2016). Our previous results have shown that infection with the H7N7 IAV resulted in loss of dendritic spines, impaired long-term potentiation in the hippocampus, and deficits in spatial memory formation, which were associated with increased numbers and activation status of microglia 30 days post-infection (dpi) well beyond the acute phase of infection (Hosseini et al., 2018). Vaccination might indeed provide the best strategy to prevent these neuropathological alterations.

In this respect, maternal influenza vaccination has been shown to promote neurogenesis and behavioral function, as well as protection against bacterial lipopolysaccharide insults in the offspring (Xia et al., 2014), and also contributes to the enhancement of hippocampal neurogenesis and working memory in pregnant mice (Qi et al., 2016). In addition, early multiple inactivated influenza vaccination exerts a positive immunomodulatory effect in the APP/PS1 Alzheimer’s disease mouse model by preventing Treg-mediated systemic immune tolerance, maintaining microglial activation, and removing *β*-Amyloid plaques, which eventually improves cognitive deficits (Yang et al., 2020). Thus, the beneficial effects of inactivated influenza vaccination on hippocampal function have in principle been suggested, at least in mouse models. This study therefore investigated the extent to which formalin-inactivated H7N7 vaccine can prevent neuroinflammation and subsequent neuronal injury induced by H7N7 infection. By this means mice were immunized three times *via* inactivated H7N7 vaccine. Fourteen days after the last vaccination, at the peak of immunity, both young and old animals were infected with H7N7 and analyzed at the peak of infection 8 days and 10 days after infection, respectively. Because IAVs cannot cause persistent infection, we hypothesized that if the onset of neuroinflammation and subsequent neuronal damage can be attenuated by vaccination during the acute phase of the disease, long-term effects and further neurological sequelae induced by IAV may also be prevented, even if the vaccination cannot fully prevent infection.

The response to vaccination seemed to be different between young and old mice, especially after the second vaccination. Oder mice did not lose as much bodyweight as young mice in the first days after vaccination. This can clearly be attributed to the different response of the young and old immune systems to vaccination. This has to be put into the context of physiological aging, which is accompanied by a decline in the normal function of the immune system, termed “immunosenescence.” This age-related decline in immunity not only leads to increased susceptibility to infection, but also reduces the prophylactic efficacy of vaccination (Lord, 2013). Accordingly, a systematic review and meta-analysis found that the effectiveness of influenza vaccination was lower in the elderly than in younger adults during various influenza seasons (Gross et al., 1995). In addition, older individuals produce antibodies more slowly compared with young adults (Ciabattini et al., 2018). Therefore, it is also assumed that older mice have lower immunity compared to young individuals, which is confirmed in different types of vaccinations (Kovacs et al., 2009).

It is worth noting that 14 days after the second immune boost, when the mice were infected with the H7N7, both the young and older cohorts showed symptoms of infection, manifested externally in a loss of bodyweight and general scoring. Interestingly, the vaccinated young mice seemed to react less upon infection and lost less weight than the unvaccinated. However, this was not the case in the older mice, here the response to infection seemed to be even stronger in the vaccinated older mice than in the unvaccinated. The changes in bodyweight after infection might depend on the secretion of proinflammatory cytokines, especially IL-1β. It has been shown that IL-1β deficient mice lost less weight than wild-type animals after injection of bacterial lipopolysaccharides (Kozak et al., 1995). Consistent with previous reports, bodyweight loss in unvaccinated animals after H7N7 infection was also associated with elevated IL-1β levels, whereas IL-1β levels were lower in the young vaccinated animals that exhibited less bodyweight loss. However, cytokine release in the lungs was high in the vaccinated old mice, sometimes slightly higher than in unvaccinated old mice, which might be associated with greater bodyweight loss. Nevertheless, survival rate in each cohort was higher in the vaccinated animals. This is consistent with clinical evidence that while influenza vaccination is less effective in the elderly than in younger adults, it reduces the risk of winter mortality from any cause by 50% in the elderly (Simonsen et al., 2005).

The results of this study also showed that H7N7 infection induced inflammatory cytokines and chemokines in the lung as a target tissue for influenza viruses. Exuberant immune responses induced after infection have been described as a “cytokine storm” associated with excessive levels of proinflammatory cytokines and widespread tissue damage. Recent studies have painted a more complex picture of cytokine networks and their contribution to clinical outcomes (Guo and Thomas, 2017).

In addition, a mirror-image response of cytokines has been demonstrated in the brains of infected mice. Based on the increased brain levels of proinflammatory cytokines IFNγ, IL1β, TNFα, and IL-6 after H7N7 IAV infection, which may be a consequence of direct neuronal infection or diffusion of cytokines from the periphery to the brain, microglia and infiltrating macrophages may be induced to exhibit proinflammatory phenotypic features (Tang and Le, 2016). This phenotype is described as the initial response to infection to defend the affected tissue. However, they also trigger neurotoxic mechanisms that crucially contribute to neuronal damage and synapse loss (Waltl and Kalinke, 2022). Because vaccination led to a decrease in proinflammatory mediators and an increase in anti-inflammatory IL-10 especially in young mice, it could alter microglia/macrophages in both the periphery and brain to exhibit protective expression patterns that promote the resolution of inflammation (Tang and Le, 2016). In the lungs, levels of the proinflammatory cytokine TNFα were actually further elevated after H7N7 infection in both young and older mice previously vaccinated. Interestingly, a previous study reported that TNFα is critical for negatively regulating the extent of lung immunopathology during acute H1N1 infection (Damjanovic et al., 2011). Moreover, another report indicated a potent anti-influenza virus effect of TNFα in lung epithelial cells (Seo and Webster, 2002). Therefore, vaccine-induced upregulation of TNFα in lung tissue after H7N7 infection may have a protective effect in the acute phase of the disease, because cytokines also play an important role in viral clearance, which has been observed in both young and older cohorts and may underlie the mechanisms of high survival rates after vaccination. However, the role of elevated TNFα levels in microglial activation and subsequent neuronal injury (Jayaraman et al., 2021) should not be neglected.

Neuronal damage after neurotropic IAV infection could be mediated by neuroinflammation in which microglia play a crucial role (Ludlow et al., 2016). An indication of the status of microglial activation is an increase in microglial density, as previously found in IAV infections. Increased microglia density was described in the hippocampus of H7N7 infected mice at 30 dpi (Hosseini et al., 2018). Moreover, H5N1 infection at 60 and 90 dpi resulted in an increase not only in the total number of microglia but also in the number of reactive microglial cells in the *substantia nigra pars compacta* (Jang et al., 2012). In the present study, an increase in microglia in the CA1 and *dentate gyrus* subregions of the hippocampus was observed in young mice as early as 8 days after infection with the H7N7, which was attenuated by vaccination. This highlights the positive preventive effect of vaccination on microglial activity triggered by H7N7 infection, which in turn may be due to cytokine and chemokine levels in the periphery and brain. However, in aged mice, a significant increase in microglial numbers was detected only in the *dentate gyrus* 10 days after infection, which may be due to the delayed, milder, but more sustained immune response triggered by IAV infection in aged mice compared with young mice (Lu et al., 2018). The most likely explanation is that the neuroinflammatory responses in aged mice sets in later but persist longer in comparison to young adult mice. Remarkably, higher numbers of microglia were detected in both subregions in aged mice after vaccination *per se*, and no further increase was evident after infection of the vaccinated mice. This may be due to inflammaging, low-grade chronic inflammation, and aging in various organs, including the brain (Gabuzda and Yankner, 2013). By this means, repeated vaccination might increase low-grade chronic neuroinflammation, leading to increased numbers of microglial cells. Other indicators of microglial activation in response to various stimuli are rapid morphological changes such as enlargement of their cell body (Nakajima and Kohsaka, 2001; Kongsui et al., 2014). Again, our data indicated that the infection triggered an enlargement of microglial cells in both subregions of the hippocampus, which was also seen in the previously vaccinated mice in both young and old cohorts, although the enlargement in the *dentate gyrus* was significantly less pronounced in vaccinated mice. This suggests that the enlargement of microglial cell bodies occurs prior to their further proliferation. The reason that the preventive effect of vaccination was more prominent in the *dentate gyrus* may be that more H7N7-induced inflammation and neuronal damage occur in this subregion because of its role in adult neurogenesis and the *dentate gyrus* being targeted by neurotropic viral attack, as described previously (Rubin et al., 1999; Heimrich et al., 2009). Therefore, the preventive effect of vaccination may also be more visible in this subregion.

Another morphological feature that provides information about the activation state of microglia is the complexity of their processes. Interestingly, in both cohorts, the number of branch points increased after infection in previously vaccinated animals, confirming a higher complexity. Thus, vaccination could potentially cause microglia to transition to a hyper-ramified morphology in response to infection, which has been observed as the initial response of microglia in various non-pathological and pathophysiological events (Beynon and Walker, 2012). This initial hyper-ramified microglial phenotype, described as environmentally alert microglia (Ziebell et al., 2015), may allow for additional surveillance following H7N7 infection in vaccinated animals, as the cells are more sensitive to the virus due to their prior contact with the inactivated virus itself or a mild inflammatory response in the brain caused by vaccination. Since microglia play a crucial role in the defense against viral infections in the CNS (Waltl and Kalinke, 2022), this may even be a protective mechanism induced by vaccination rather than a pathological change, and a lower magnitude in older mice may again confirm the slower and more sustained immune response induced by influenza in the elderly (Lu et al., 2018).

Loss of dendritic spines in hippocampal neurons may be one mechanism of how immediate and long-term cognitive consequences are mediated after IAV infection. Previously, acute loss of dendritic spines in hippocampal neurons 7 days after H1N1 infection was found to be associated with deficits in spatial learning (Jurgens et al., 2012). Furthermore, our previous findings demonstrated impairments in learning and memory formation 30 days after H7N7 infection in mice, which were associated with significant loss of dendritic spines in all hippocampal subregions (Hosseini et al., 2018). Consistent with previous studies, examination of hippocampal neuron morphology in both cohorts revealed reduced dendritic spine density caused by H7N7 infection in *dentate gyrus* neurons, which was not seen in previously vaccinated mice. These results suggest a preventive effect of vaccination against hippocampal spine loss induced by H7N7 infection, such that vaccination may also prevent subsequent long-term cognitive impairment resulting from infection. Nevertheless, as mentioned above, the reduction in spine density was more pronounced in the *dentate gyrus*, which may be due to the attraction of this subregion for neurotropic viruses (Rubin et al., 1999; Heimrich et al., 2009). However, the neuroprotective role of the inactivated influenza vaccine might counteract the loss of spines induced by viral infection. Indeed, influenza vaccine has been shown to lead to the recruitment of T lymphocytes from the periphery to the choroid plexus in mice, which promote increased levels of brain-derived growth factor (BDNF) and insulin-like growth factor-1 (IGF-1) in the hippocampus in communication with resident cells, whereas these effects were abolished by anti-TCR antibody treatment (Qi et al., 2016). These neurotrophic factors have been shown to promote synaptogenesis (McCusker et al., 2006) and synaptic plasticity (Korte et al., 1995; Zagrebelsky and Korte, 2014).

Mechanistically, the loss of synapses that may contribute to cognitive dysfunction after viral infection could be mediated by excessive microglial synaptic pruning, which has been observed in both physiological and pathological brain conditions (Paolicelli et al., 2011; Kettenmann et al., 2013). Microglia-mediated pruning of synaptic compartments has been observed in several neurotropic viral infections such as West Nile virus and Zika virus (Vasek et al., 2016; Garber et al., 2019). Therefore, this event could also be considered as an underlying mechanism of neuronal damage caused by H7N7 infection, which might be attenuated after vaccination. Our results provided evidence that after H7N7 infection, the engulfment of Homer-1-labeled excitatory postsynaptic terminals into lysosomal compartments of microglial cells increased to some extent in both subregions of the hippocampus of young mice, but more strongly in the *dentate gyrus* of older mice, which may reflect the reduced dendritic spines in this subregion in both cohorts, as Homer-1 is a postsynaptic density (PSD) scaffold protein involved in synaptic plasticity (Tao-Cheng et al., 2014). A remaining open questions is why the increased uptake of Homer-1 in microglial lysosomes in the CA1 subregion is not reflected in the analysis of dendritic spine density in young mice. One likely explanation for this scenario is that the increased uptake of Homer-1 terminals into microglial lysosomes and the decreased density of dendritic spines are not two simultaneous events in the cells.

In addition, it is important to note that mechanisms other than direct microglial engulfment of synaptic compartments may be involved in the loss of dendritic spines induced by viral infection, e.g., increased levels of proinflammatory cytokines and subsequent neuronal excitotoxicity (Galic et al., 2012).

In addition to the important role of microglia in this scenario, astrocytes are also crucial players in the neuroinflammatory response (Wilhelmsson et al., 2006). Interestingly, our results showed that the intensity of GFAP fluorescent decreased in the young unvaccinated infected groups, possibly due to the infection of astrocytes by neurotropic influenza viruses, which has been previously reported to lead to apoptosis (Pei et al., 2014; Ng et al., 2018; Hosseini and Korte, 2023). In this context, it has also been shown that apoptosis of astrocytes in the hippocampus with activated caspase-3 leads to a sharp decrease in astrocyte number (Kim et al., 2022). Moreover, this evidence is consistent with previous findings that GFAP was downregulated during acute corneal HSV-1 infection and upregulated in the late stage, suggesting that GFAP may play some role during corneal HSV-1 infection (Zhao et al., 2014). Downregulation of GFAP was also found in several cell lines infected with neurotropic human cytomegalovirus (Lee et al., 2005). Therefore, astrocytes may also play an important inflammatory role during H7N7 IAV infection, especially in young mice, which can be modulated by vaccination. However, in the older animals, a slight decrease in GFAP expression was observed in both vaccinated mice and unvaccinated infected animals but was not statistically significant. This may suggest that astrocytes undergo age-dependent changes in gene expression and susceptibility to pathogens (Palmer and Ousman, 2018), warranting further investigation.

Taken together, these results shed mechanistic insight on the beneficial effects of vaccination for the neurological consequences of H7N7 infection, although these effects were different in young and old animals. Vaccination always had a better effect in young mice, which may be attributed to immunosenescence and “inflammaging” in older animals. Although initial disease symptoms and some neuroinflammatory aspects were higher in older than in young mice, survival and even detailed synaptic loss induced by infection were reversed in previously vaccinated mice. Therefore, vaccination is suggested as a good intervention to combat the consequences of the virus, especially on the nervous system, as it is a very important part of the body that controls many important functions.

Further exploration of the mechanisms underlying the potential beneficial effects of vaccination with different types of influenza vaccines against neuroinflammation and the subsequent deleterious effects of IAV infection on neuronal structure and function may pave the way for the use of vaccination as a potential strategy to prevent neurological sequelae caused by IAV infection in the future.

## Data Availability

The raw data supporting the conclusion of this article will be made available by the authors, without undue reservation.
